# Bioinformatics network analyses of growth differentiation factor 11

**DOI:** 10.1515/biol-2022-0044

**Published:** 2022-04-26

**Authors:** Feng Zhang, Xia Yang, Zhijun Bao

**Affiliations:** Huadong Hospital Affiliated to Fudan University, 221 West Yan’an Road, Shanghai, 200040, China; National Clinical Research Center for Aging and Medicine, Huashan Hospital, Fudan University, 12 Mid Urumqi Road, Shanghai, 200040, China; Shanghai Key Laboratory of Clinical Geriatric Medicine, 221 West Yan’an Road, Shanghai, 200040, China; Department of Integrative Biology and Physiology, University of California, Los Angeles, 610 Charles E. Young Dr. E, Terasaki Life Sciences Bldg. Rm 2000B, Los Angeles, CA90095, USA; Department of Geriatrics, Huashan Hospital Affiliated to Fudan University, 12 Mid Urumqi Road, Shanghai, 200040, China

**Keywords:** GDF11, senescence, apoptosis, DNA repair, telomere maintenance

## Abstract

Growth differentiation factor 11 (GDF11) has been implicated in rejuvenating functions in age-related diseases. The molecular mechanisms connecting GDF11 with these anti-aging phenomena, including reverse age-related cardiac hypertrophy and vascular and neurogenic rejuvenation, remain unclear. In this study, we sought to uncover the molecular functions of GDF11 using bioinformatics and network-driven analyses at the human gene and transcription levels using the gene co-expression network analysis, the protein–protein interaction network analysis, and the transcription factor network analysis. Our findings suggested that GDF11 is involved in a variety of functions, such as apoptosis, DNA repair, telomere maintenance, and interaction with key transcription factors, such as MYC proto-oncogene, specificity protein 1, and ETS proto-oncogene 2. The human skin fibroblast premature senescence model was established by UVB. The treatment with 10 ng/mL GDF11 in this cell model could reduce cell damage, reduce the apoptosis rate and the expression of caspase-3, and increase the length of telomeres. Therefore, our findings shed light on the functions of GDF11 and provide insights into the roles of GDF11 in aging.

## 1 Introduction

Aging is a worldwide health issue. According to a report published by the Department of Economic and Social Affairs, Population Division, United Nations, the number of people aged 65 years or over was 727 million worldwide in 2020, with the global elderly population increasing and estimated to reach 1.5 billion by 2050 [1]. Aging involves not only changes in appearances, such as the development of wrinkles or gray hair, but also the hypofunction of organs and tissues. Cell senescence is the fundamental reason underlying age-related dysfunction [2]. The goal of geriatric research is to identify solutions to reverse cell senescence and decrease age-related disorders.

In 2013, Loffredo et al. identified growth differentiation factor 11 (GDF11) as a key factor in the rejuvenation of age-related heart hypertrophy, making GDF11 a prime candidate for reversing age-related diseases [3]. Specifically, the authors found that administering GDF11 for 4 weeks led to the normalization of enlarged cardiac myocytes, increased myocardial contractility, and decreased heart failure index, indicating heart rejuvenation at both physiological and functional levels. Additional studies have supported these findings, showing evidence for the role of GDF11 in rejuvenating the vascular, nervous, and skeletal systems [4–7]. GDF11 is a member of the transforming growth factor (TGF)-β superfamily and is known to play an important role in development by maintaining a balance between proliferation and differentiation. GDF11 can arrest the cell cycle at the G1 phase through p21cip1/p27kip1 and induce differentiation of cells into maturation [8], thereby controlling the number of mature cells. However, these processes fail to explain the role of GDF11 in rejuvenation. To date, the exact mechanisms by which GDF11 rejuvenates organs and reverses aging remain unclear. Our goal was to implement a bioinformatics- and network-driven analysis approach to understand the role of GDF11 in aging and anti-aging in biological systems to better improve global age-related diseases. Bioinformatics analysis is a useful method to study biological molecules that have unclear functionalities, which has the potential to reveal novel functions of specific genes and related or similar molecules.

The bioinformatics methods used in this study included gene co-expression network analysis, protein–protein interaction network analysis, and transcription factor network analysis. These powerful methodologies can help reveal the functions of GDF11. In addition, a human skin fibroblast (HSF) premature senescence model was established by UVB, followed by the treatment with 10 ng/mL GDF11 to figure out the role of GDF11 in cell damage, apoptosis rate as well as the length of telomeres.

## 2 Materials and methods

### 2.1 Identification of functional modules involving GDF11

To explore the molecular functions of GDF11, we first extracted functional modules that contain GDF11, as determined using various approaches. A functional module is defined as a group of molecules that share similar functions or are involved in a common biological process [9]. Exploring functional modules could expand the research power from a targeted molecule, such as GDF11, to a variety of related molecules, thus providing a further understanding of the functional roles of GDF11 and uncovering correlated genes involving a target gene. To this end, we used (1) tissue-specific gene co-expression network analysis, (2) protein-protein interaction network analysis and (3) transcription factor network analysis.

### 2.2 Gene co-expression networks

Gene co-expression networks are statistics based on networks that capture genes from different microarray experiments [9], and the co-expressed genes are functionally related. It is a practical method to uncover genes whose functions are still unknown and provides insight into the gene function in certain specific biological processes [10]. We used weighted gene co-expression network analysis (WGCNA) [11] to construct gene co-expression networks. This is a widely used machine algorithm for selecting modules and constructing gene co-expression networks. Numerous studies have demonstrated the effectiveness of WGCNA in building functional biological modules [12,13].

### 2.3 Code availability

The WGCNA R routine source is available at https://horvath.genetics.ucla.edu/html/CoexpressionNetwork/Rpackages/WGCNA/Tutorials.

Using WGCNA, we constructed human co-expression networks of various tissues using gene expression data from the Genotype-Tissue Expression project (GTEx) [14]. GTEx is the most comprehensive tissue-specific transcriptomic and genetic dataset derived from human tissues, providing a platform to understand the relationships between genetic variation and gene expression. GTEx consists of whole-genome DNA sequence and RNA-sequencing data from nearly 1,000 deceased adult donors and 53 tissue sites (e.g., brain, liver). We used the human gene co-expression networks built by WGCNA using GTEx RNA-sequencing data to extract genetic co-expression modules that contain GDF11. The GTEx data that we applied and downloaded are the gene-level transcript per million data as GTEx_Analysis_2016-01-15_v7_RNASeQCv1.1.8_gene_tpm.gct.gz from GTEx official website https://gtexportal.org/home/datasets. We present analyses of the v7 data, examining 10,294 RNA-sequencing samples from 48 tissues of 620 postmortem donors. We performed several steps of preprocessing for data normalization as the WGCNA website suggests (https://horvath.genetics.ucla.edu/html/CoexpressionNetwork/Rpackages/WGCNA/faq.html): log-transforming the data using log2(*x* + 1) as variance-stabilizing transformation and removing features whose counts are consistently low (removing all features that have a count of <1 in >60% of samples).

### 2.4 Protein–protein interaction network

To explore the protein level functions of GDF11, we used human protein–protein interaction networks from the Search Tool for the Retrieval of Interacting Genes/Proteins (STRING) database [15]. The STRING database is a functional protein association network composed of physical and functional interactions [15]. We searched GDF11 on the STRING website (https://www.string-db.org) in Homo sapiens with a confidence score of above 0.7. The confidence score of GDF11 and the interaction protein are presented in Table S4. Through these analyses, we can know which proteins have direct and indirect contacts with GDF11 and understand more about the molecular activities of GDF11 in biochemical cascade context.

### 2.5 Transcription factor network

We used the functional analysis of the mammalian genome 5 (FANTOM5), a comprehensive analysis of gene regulation in different cell types to analyze the transcriptomic network of GDF11. FANTOM5 provides an overall map of gene activity across the human body as well as a holistic view of the complex networks that regulate gene expression across a wide variety of cell types [16]. The FANTOM5 networks are provided as tab-separated text files with three columns (column 1: transcription factor gene, column 2: target gene, and column 3: edge weight) [17]. We searched GDF11 in the second column and obtained its related transcription factors and their weighting value at the FANTOM5 official website http://fantom.gsc.riken.jp.

### 2.6 Annotation of GDF11 modules using functional enrichment analysis

After identifying co-expressed genes and relevant *GDF11* transcription factors from each functional module, we used gene set enrichment analysis (GSEA) [18]. Using GSEA (website http://software.broadinstitute.org/gsea/index.jsp), we annotated the enriched biological pathways shared by genes in the same module, which provided us with insight into GDF11 functions. The annotated pathways were collected from BioCarta, KEGG, and Reactome databases. Pathways reaching *P* ≤ 0.05 with a false discovery rate (FDR) ≤0.01 were collected. Overlaps with shared gene numbers ≥3 and fold enrichment involving the top 30% were considered significant pathways [19].

### 2.7 Visualization of analysis results

Subsequently, Cytoscape [20] (https://cytoscape.org) was used to further investigate the molecular relationships involving protein and transcription factor modules, and pinpoint key regulators.

### 2.8 Cell culture and modeling

HSFs were purchased from the Website of Microorganism for Search (bio-51608, China, Beijing), and the cells were cultured in a dulbecco’s modified eagle medium (DMEM) (11965092, Gibco, New York, USA) containing 10% fetal bovine serum (FBS, 10099141; Gibco, New York, USA) in a cell incubator at 37°C with 5% CO_2_. HSFs in the logarithmic division period were detached and resuspended and then cultured in a 6-well plate at a cell density of 1 × 10^5^ cells/well for cell climbing (treated with gelatin in advance). The cells were divided into three groups, namely a control group, a Model group, and a Model + GDF11 group, with three wells in each group.

When the cells grew to 80% confluence, the cells in the Model group and the Model + GDF11 group were irradiated with UVB at a dose of 10 MJ/cm^2^ for 5 consecutive times. Cells in the control group were not treated. After cell culture for 24h, the culture medium in the control group and the Model group were replaced with DMEM supplemented with 10% FBS, and the Model + GDF11 group was replaced with DMEM containing 10% FBS and 10 ng/mL GDF11 (SRP4576-10UG; Merck, Shanghai, China). After 48 h of culture, the morphology of the cells was observed under a microscope, and the apoptosis and telomerase length were measured.

### 2.9 Terminal deoxyribonucleotide transferase-mediated deoxyuridine triphosphate-digoxigenin nick end labeling (TUNEL) staining

The TUNEL staining kit was purchased from Nanjing Novozan Biotechnology Co., Ltd. (A113-03, Nanjing, China), and the apoptosis of HSFs was measured according to the kit instructions. Specifically, the cells were fixed with pre-cooled 4% paraformaldehyde for 25 min, incubated with 20 μg/mL of Proteinase K solution for 20 min, and then added with 100 μL of 1× equilibration buffer to cover the cells to be tested. Upon 15-min incubation, the cells were added with TdT buffer and incubated at 37°C for 60 min. Finally, the nuclei were counter-stained with 4′,6-diamidino-2-phenylindole 2HCI for 5 min. Then, the cells were blocked and observed under a microscope.

### 2.10 Western blot

The cells were lysed with lysis buffer, and the total protein concentration was determined with the BCA Protein Detection Kit (P0010; Beyotime Biotechnology, Shanghai, China). Then, the protein samples were diluted with 5× sample buffer, separated by electrophoresis in a 12% separation gel for 90 min, and incubated with TBST blocking solution containing 5% (w/v) skimmed milk powder for 1 h at room temperature. Next, the cells were combined with primary antibodies against B-cell lymphoma 2 (Bcl-2) (1:1,000, ab32124; Abcam, Cambridge, UK), caspase-3 (1:5,000, ab32351; Abcam), glyceraldehyde phosphate dehydrogenase (GAPDH, 1:1,000, AB-PR 001; Hangzhou Xianzhi Biological Co., Ltd., Zhejiang, China) and incubated overnight at 4°C. Afterward, the membrane was washed and incubated at room temperature for 1 h with the secondary antibody (1:500, BA1054; Wuhan Boster Bioengineering Co., Ltd., Wuhan, Hubei, China), and the BioSpectrum Imaging System chemiluminescence analysis imaging system (UVP, USA) was used for photographing.

### 2.11 Reverse transcription quantification polymerase chain reaction (RT-qPCR)

Referring to the manufacturer’s instructions, the total RNA sample was extracted from HSFs using TRIzol reagent (15596-026; Ambion, Shanghai, China). PrimeScript RT Reagent Kit (3733; TaKaRa, Japan) was utilized to synthesize 1 μg of total RNA into cDNA. HiScript^®^ II Q RT SuperMix for qPCR (+gDNA Wiper) Kit (R223-01; VAZYME, Nanjing, China) and Bio-Rad CFX384 Touch Detection System were used for real-time PCR. After the reaction system was pre-denatured at 95°C for 10 min, it was denatured at 95°C for 15 s, annealed and extended at 60°C for 1 min, and amplified for 40 cycles. All reactions were repeated three times. GAPDH was the internal reference gene of Bcl-2 and caspase-3, and 36B4 was the internal reference gene of the telomere. The data were corresponded to the average value of 2^−ΔΔCt^ from at least three independent experimental operations and were normalized with the internal reference gene. Data analyses were performed with the 2^−ΔΔCt^ method, and ΔΔCt = experimental group (Ct target gene − Ct internal control) − control group (Ct target gene − Ct internal control). The primers used for amplification are listed in Table 1. Telomere fragment amplification is *T* reaction, 36B4 fragment amplification is *S* reaction, relative *T/*S ratio is 2^−ΔΔCt^, and telomere length (Kb) is 1.585 × *T/S* + 3.582.

**Table 1:** RT-qPCR primers used for amplification

Name	Primer	Sequence	Size (bp)
Homo GAPDH	Forward	5′-TCAAGAAGGTGGTGAAGCAGG-3′	115
	Reverse	5′-TCAAAGGTGGAGGAGTGGGT-3′	
Homo BCL2	Forward	5′-GCCTTCTTTGAGTTCGGTGG-3′	192
	Reverse	5′-GAAATCAAACAGAGGCCGCA-3′	
Homo Caspase3	Forward	5′-ACTGGACTGTGGCATTGAGA-3′	162
	Reverse	5′-GCACAAAGCGACTGGATGAA-3′	

## 3 Results

### 3.1 Gene co-expression network analysis

Using gene co-expression networks, we found GDF11 to be part of 23 modules (see Experimental Procedures 2.1) from 11 different systems or organs (Table S1). We analyzed the biological pathways of each GDF11-containing module (Table S2) to determine the general functions of GDF11, as summarized in Table 2.

**Table 2:** Top functions of GDF11-containing genetic modules in human co-expression networks

Tissue	Top functions*
Adipose	Apoptosis, DNA repair, telomere maintenance, transcription regulation, proliferation
Nervous system	Apoptosis, DNA damage checkpoint, axon guidance, insulin/IGF-1 pathway, PI3K/AKT pathway, NCAM signaling, expression, immune, metabolism
Cardiovascular system	Apoptosis, DNA repair, telomere maintenance, GPCR pathway, expression, metabolism
Digestive system	Apoptosis, biological oxidation, ER stress, ERK/MAPK pathway, DAG/IP3/Ca^2+^ pathway, expression, metabolism
Liver	NOTCH pathway, collagen formation, NCAM signaling, extracellular matrix
Lung	Ion transport, metabolism
Kidney	Extracellular matrix
Skeletal muscle	Apoptosis, ERK/MAPK pathway, toll-like receptor pathway, muscle contraction, circadian rhythm
Endocrine system	Apoptosis, DNA repair, PIK3/AKT pathway, axon guide, metabolism
Female reproduction system	Translation regulation, expression
Male reproduction system	Apoptosis, IGF signaling, transcription regulation, proliferation, metabolism

*Top functions: the functions were selected and summarized from significant pathways (*P* ≤ 0.05, FDR ≤ 0.01, shared gene numbers ≥3 and fold enrichment at top 30%) and listed in no particular order. Detailed data are shown in Table S2.

The common functions of GDF11 are mostly related to apoptosis, DNA repair, telomere maintenance, certain key pathways (including PI3K/AKT, ERK/MAPK, DAG/IP3/Ca^2+^, toll-like receptor, and GPCR pathways), transcriptional regulation, cell proliferation, and metabolism. However, endoplasmic reticulum (ER) stress, collagen formation, axon guide, ion transport, and circadian rhythm were found in some specific tissue types. In Table 2 and Table S2, we have summarized the main age-related functions of GDF11 gene co-expression modules in each tissue and system.

### 3.2 The implication of GDF11 in apoptosis

Apoptosis is the most common function among systems resulting from our data analyses and plays a major role in aging and age-related diseases. We observed GDF11 in caspase cascade-related apoptotic cleavage in the visceral adipose, hippocampus, cerebellar hemisphere, left ventricle, terminal ileum of the small intestine, skeletal muscle, pituitary gland, and prostate tissues.

### 3.3 The implication of GDF11 in DNA repair

DNA damage increases with aging and declining DNA repair, leading to cell senescence and even cell death. We observed GDF11 as potentially beneficial for DNA repair in many aspects, including single-stranded DNA damage repair (global genomic-nucleotide excision repair [GG-NER], base excision repair [BER], and DNA mismatch repair [MMR]) and double-stranded break repair (homology-directed repair [HDR] and nonhomologous end-joining [NHEJ]). In the adipose tissue, we found that the GDF11 module was involved in single- and double-stranded DNA repair by GG-NER, BER, MMR, and HDR. Double-stranded break repair is also found in the left ventricle, involving HDR and NHEJ, whereas only BER is involved in the pituitary. Moreover, the GDF11 module functions as a DNA damage checkpoint in the tibial nerve.

### 3.4 The involvement of GDF11 in telomere maintenance

Telomeres and telomerases are likely to play roles in circadian biological clocks involving cellular replicative senescence. Previous studies have focused primarily on telomere maintenance and slowing the aging process [21]. Our results indicate that the gene co-expression module of GDF11 is involved in telomere extension in adipose tissue and has been associated with telomeres, telomerase, cellular aging, and immortality in the left ventricle.

Genetic co-expression network studies surrounding GDF11 have also implicated GDF11 in relieving ER stress caused by aging in the NCAM signaling pathway, which could benefit cell survival and regeneration; in the toll-like receptor pathway for necroptosis, which could help to clear aging cells; in the PI3K/AKT signaling for proliferation; and lastly, in the MAPK/ERK signaling for proliferation and angiogenesis. Our findings involving apoptosis, DNA repair, telomere maintenance, and other GDF11 functions may help explain the role of GDF11 in reversing age-related diseases.

### 3.5 Interaction between GDF11 and proteins

**Figure 1:**
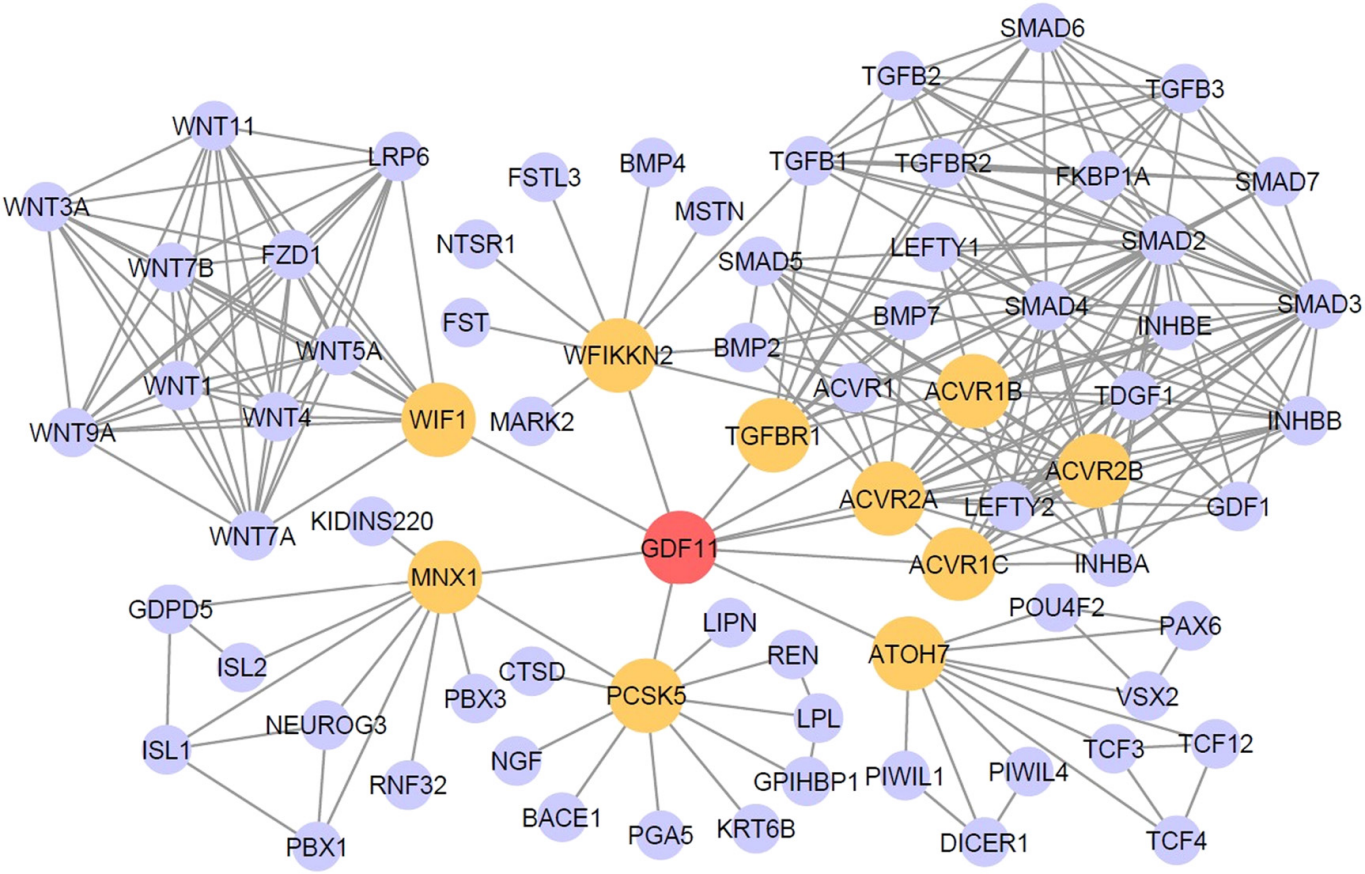
Protein–protein interaction network of GDF11 in STRING network visualizing GDF11-interactive neighbor proteins. Yellow nodes represent the first-layer interactive protein of GDF11 and lavender nodes represent the second-layer neighbor proteins. The gray lines represent the correlation of the proteins.

Using the protein networks from the STRING databases, we searched GDF11 and its first (yellow) and second (lavender) layer neighboring proteins and visualized the protein networks using Cytoscape (Figure 1).

The GDF11 STRING network in Figure 1 shows that, besides the generally known receptors of GDF11 (ACVR1B, ACVR1C, ACVR2A, ACVR2B, and TGFBR1), the first layer neighbors of GDF11 also include WIF1, MNX1, PCSK5, ATOH7, and WFIKKN2. WIF1 is involved in Wnt signaling, which is an important pathway controlling tissue regeneration in adults [22]. MNX1 is involved in the neural stem cell differentiation pathway and regulation of the beta-cell development pathway [23,24]. PCSK5 plays an essential role in pregnancy establishment by proteolytic activation of several important factors [25]. ATOH7 is a transcription factor involved in the differentiation of retinal ganglion cells [26]. WFIKKN2 is one of the inhibitors of GDF11 [27].

### 3.6 Interaction between GDF11 and transcription factors

**Figure 2:**
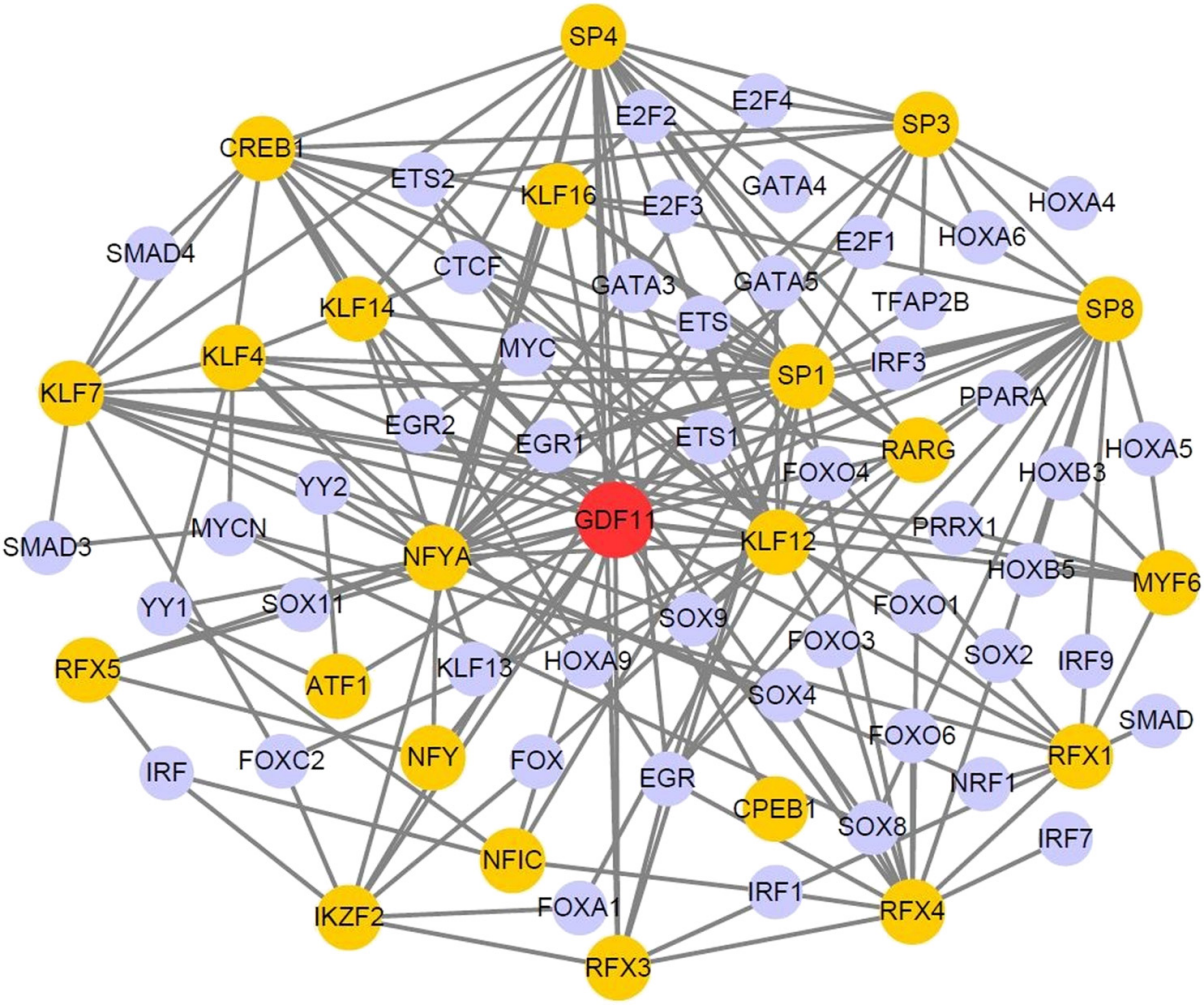
FANTOM5 neural stem cell transcription factor network involving GDF11. Visualization of *GDF11*-interactive transcription factors in neural stem cells. Yellow nodes represent the first-layer interactive factor of GDF11 and lavender nodes represent the second-layer neighbor factors. The gray lines represent the correlation of the transcription factors.

We used FANTOM5 [16], a comprehensive tool for analyzing gene regulation in different cell types, to determine the transcriptomic network around GDF11 in different adult cell types. We found GDF11 transcriptomic networks in 76 cell types across 10 human systems (nervous, cardiovascular, digestive, respiratory, urinary, skeletal muscle, endocrine, immune system, and female and male reproductive systems). We observed an interesting phenomenon in that the transcription factors were similar among the different cell types (Table S3). As an example, we analyzed the largest transcriptomic network involving GDF11 in neural stem cells (Figure 2). Yellow nodes represent the first-layer GDF11 neighbors, and lavender nodes indicate the second-layer neighbors.

From our data, we concluded that the most common transcription factor neighbors of GDF11 are cAMP response element-binding protein 1 (CREB1) and nuclear transcription factor Y subunit alpha (NFYA), which is identified in 93.42% of cell types (Table S3). These findings suggested that CREB1 and NFYA are highly conserved and related to the most common function of GDF11 across cell types and systems. CREB1 connects GDF11 with CTCF (a transcriptional repressor of MYC proto-oncogene [MYC] [28]), ETS proto-oncogene 2 (ETS2, reported as a regulator of telomerase [29]), SMAD4 (a member of GDF11 downstream pathway), and the first-layer SP/KLF family (a transcription factor family that maintains stem cell proliferation and differentiation [30]). NFYA is a post-transcriptional regulation factor that is also connected with the SP/KLF family. These findings emphasize the importance of GDF11 and related transcription factors in apoptosis, DNA repair, telomere maintenance, and cellular proliferation and differentiation, which are associated with longevity. Finally, we studied the forkhead box (FOXO) family, homeobox family, regulatory factor X family, E2F transcription factor family, and certain other longevity-related factors in this transcriptomic network.

### 3.7 GDF11 inhibits HSF premature senescence

**Figure 3:**
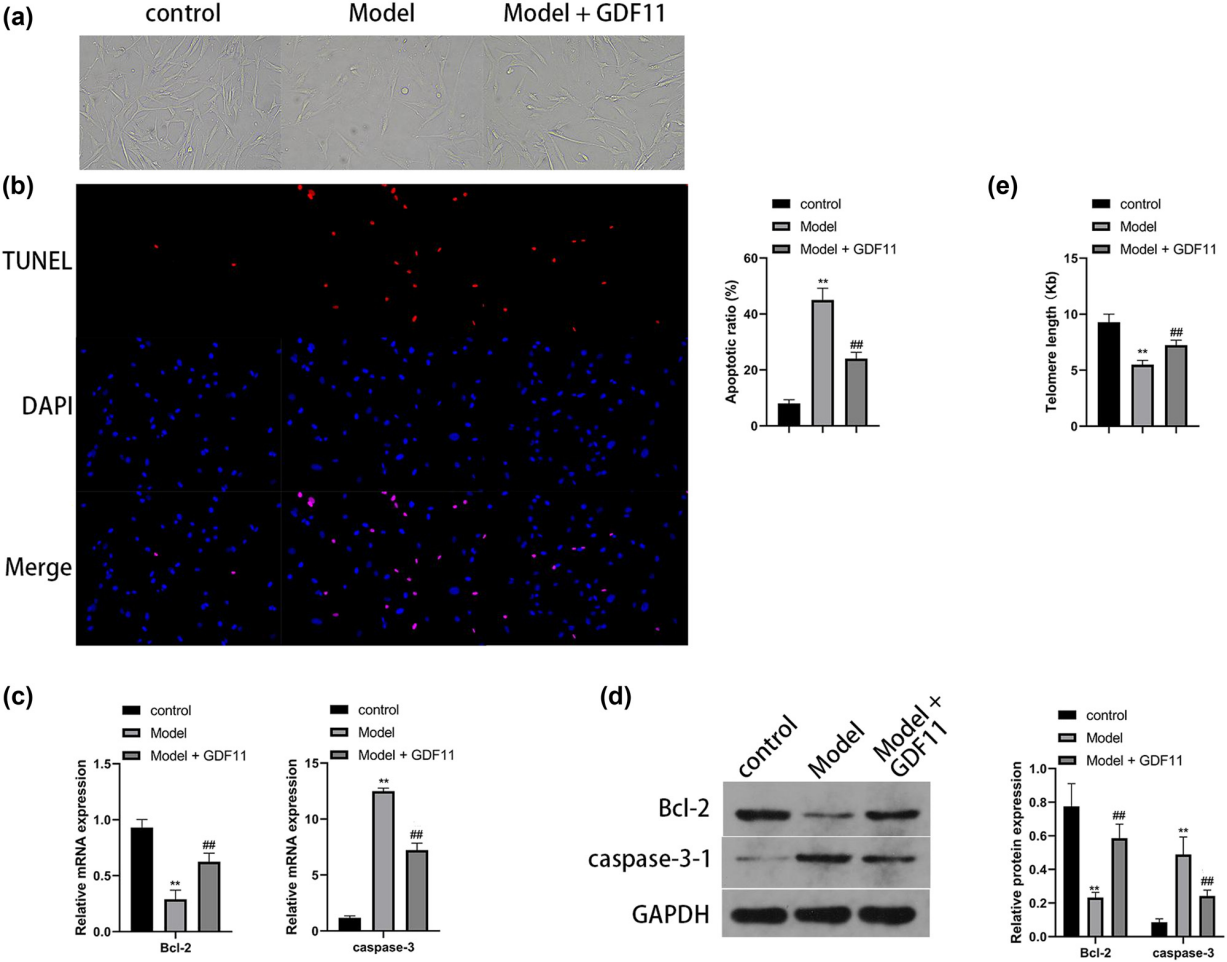
GDF11 inhibits HSF premature senescence. The HSFs were cultured *in vitro*, cells were given UVB radiation of 10 MJ/cm^2^ for five consecutive times, cells were treated with or without 10 ng/mL GDF11. (a) The morphology of skin fibroblasts in each group was observed under optical microscope; magnification is 100 times. (b) The TUNEL assay was used to detect cell apoptosis in each group; blue represents the DAPI, and red represents the TUNEL-positive cells. (c) qPCR was used to detect the mRNA levels of bcl-2 and caspase3 in each group. (d) The protein levels of bcl-2 and caspase3 were determined by Western blot. (e) The telomere length of each group was detected by qPCR. ** represents *P* < 0.01 vs. the control group; ## indicates *P* < 0.01 when compared to the model group.

To further explore the role of GDF11 in the premature senescence of cells, we established an HSF premature senescence model and treated it with GDF11, followed by detection of the cell-related indicators. The cell morphology of each group is shown in Figure 3a. Compared with the cells in the control group, the reduced cell density, enlarged cell volume, flat shape, apparent nucleus, and vacuoles in some cells were observed in the Model group, while no significant change was found in the cell morphology of the Model + GDF11 group. Next, we detected the apoptosis rate and the expression of apoptosis-related proteins. The results indicated that in contrast to the control group, the increased apoptosis rate decreased the expression of Bcl-2 and increased the expression of caspase-3, which were found in the Model group. On the contrary, the decreased apoptosis rate increased the expression of Bcl-2 and decreased the expression of caspase-3, which were observed in the Model + GDF11 group versus the Model group (Figure 3b–d, *P* < 0.01). Furthermore, RT-qPCR was carried out to detect the telomere length of cells in each group (Figure 3e, *P* < 0.01). The findings revealed that the telomere length of cells in the control group, the Model group, and the Model + GDF11 group was 9.274 ± 0.732, 5.489 ± 0.387, and 7.236 ± 0.449, respectively. Based on the above experimental results, we could conclude that GDF11 can effectively alleviate the premature senescence of HSFs induced by UVB.

## 4 Discussion

Previous studies have shown that GDF11 may reverse age-related heart hypertrophy, improve nervous system function, and increase angiogenesis in aging animals [3,4]. During embryonic stages, GDF11 inhibits proliferation and promotes differentiation of differentiable cells [8]. However, the functions of GDF11 in adults, especially its role in reversing age-related diseases, remain poorly understood. One potential mechanism could involve GDF11 interactions with FOXO family members and inhibit hypertrophy [3]. Our network analyses also confirmed the involvement of the FOXO family in GDF11 functions in transcriptional networks. Despite this possibility, understanding of the mechanisms underlying the connection between GDF11 and rejuvenation is limited. Using our integrative network analyses, we aim to expand our knowledge of GDF11 and its functions in adulthood.

Based on our bioinformatics analyses of gene co-expression networks, protein–protein interaction networks, and transcription factor networks, we found that GDF11 is involved in apoptosis, DNA repair, telomere maintenance, transcriptional regulation, cell survival, angiogenesis, and cellular regeneration. We also identified potential novel-related transcription factors of GDF11, including MYC, SP family, CREB1, and ETS2. In the subsequent sections, we have discussed the common and novel functions uncovered by our GDF11 network analyses in detail.

Based on the human genetic data analysis, apoptosis is the most common function of GDF11 among systems (adipose tissue, nervous, cardiovascular, digestive systems, skeletal muscle tissue, and endocrine systems). Apoptosis plays a significant role in longevity [31]. Along with aging, the reactive oxygen and nitrogen intermediates, lipid peroxidation products, and advanced glycation end products will accumulate in the cells and cause apoptosis [32]. Moreover, the age-related aggrandizement of apoptosis will enhance the degree of deterioration of the function of organs and tissues in aging mammalians [33]. Furthermore, in the following experiments, we found that GDF11 could reduce the apoptosis rate of premature HSF (Figure 3). Apoptosis exists during the whole life process, and it is very important in healthy aging. However, if apoptosis is dysregulated, premature senescence-associated diseases will likely appear [34]. Zhang et al. also found that treatment of endothelial progenitor cells (EPCs) with recombinant GDF11 attenuated EPC dysfunction and apoptosis [35]. Xiao et al. demonstrated that GDF11 could alleviate intracerebral hemorrhage-induced neurological deficits, neuronal apoptosis, and inflammatory reaction [36]. In another study, Mei et al. showed that GDF11 treatment could improve diabetes-induced retinal apoptosis, capillary degeneration, and inflammation in mice, and the mechanism associated with TGF-β/Smad2, PI3k-Akt-FoxO1 activation, and NF-KB pathway inhibition [37]. In addition, in an interesting study, Delgado et al. compared platelet-rich plasma (PRP) from young (20‒25 years) and elderly (65‒85 years) donors in terms of reducing neural progenitor cell apoptosis, the effect of the Young PRP was more pronounced [38]. The studies all gave the same results as this report that GDF11 could benefit aging in reducing apoptosis rate and cell dysfunction. Next, we want to testify the signaling pathway in the process of GDF11-reducing apoptosis. Our results suggested the involvement of GDF11 in intrinsic apoptosis with SMAD/CTCF/MYC signaling. The classical pathway of GDF11 is through SMADs, which act by regulating diverse biological effects by partnering with various transcription factors [39]. The MYC inhibitor CTCF is one of the GDF11/SMAD downstream transcription factors [28] and appears in our GDF11 transcription network with MYC. MYC has recently been linked to longevity [40]. In the future, we tend to test the pathway and make it a potential treatment target for aging-related diseases.

Our results indicate that GDF11 is also involved in DNA damage repair and DNA damage checkpoints. DNA damage occurs constantly in human cells and can be attributed to environmental factors, such as UV or X-ray exposure. Extensive and cumulative DNA damage results in cell carcinogenesis, cell death, or apoptosis. Along with aging, the rate of DNA repair decreases, and a large amount of DNA damage occurs. In this case, age-related diseases are prone to occur, which can, sometimes, even cause cancer [41]. Further evidence is that several premature senility syndromes have potential DNA-repair deficiencies [42]. In this study, we observed that GDF11 was involved in DNA repair in many tissue systems, such as the adipose tissue and nervous, cardiovascular, and endocrine systems. Among these systems, GDF11 is involved in DNA damage repair checkpoint and single- or double-stranded break repair signaling, suggesting that these DNA repair mechanisms are related to PI3K/AKT signaling and specificity protein 1 (SP1), a transcription factor associated with GDF11 at the transcriptional level. Many studies have shown that the PI3K/AKT signaling pathway directly responds to DNA damage and that several downstream PI3K/AKT signaling proteins guide cell cycle checkpoint activation, DNA repair, and activation of apoptosis after unsuccessful repair [43,44]. SP1 is a promoter-binding protein and a PI3K/AKT downstream molecule. Most genes have multiple SP1 sites in the proximal promoter region. Studies have shown that the consumption of SP1 renders cells sensitive to DNA damage, decreases repair rate, and increases double-strand DNA damage frequency [44]. Beishline et al. found that SP1 appears in DNA damage regions 7.5 min after DNA damage and persists at DNA break sites for at least 8 h and that the consumption of SP1 inhibits the repair of DNA breaks [45]. From our results involving gene and transcriptional analyses in this study, GDF11 may be involved in detecting and repairing DNA damage through the SMADs/SP1 and PI3K/AKT signaling pathways. With the successful repair of DNA damage, the organism maintains a stable internal environment. Normal cell proliferation and function are conducive to the recovery of organ function in aging tissues.

Our results also link GDF11 with telomere function. Telomeres are regions of repetitive nucleotide sequences at each end of a chromosome, which protect the end of the chromosome from deterioration or fusion with neighboring chromosomes. During chromosome replication, the enzymes that duplicate DNA cannot continue their duplication to the end of a chromosome. Thus, in each replication, the end of the chromosome is shortened, which has been associated with aging. The length of telomeres has become a symbol of longevity, and telomere shortening is associated with age-related diseases. Therefore, prolonging telomere length has become a significant research direction to delay aging. Our research suggests that GDF11 can upregulate telomeres by regulating telomerase reverse transcriptase (TERT) through the transcription-related factors SP1, ETS2, and GDF11/SMAD/CTCF/MYC signaling pathways. Telomerase acts as reverse transcriptase in telomere elongation. TERT is a catalytic subunit of telomerase, which, together with the telomerase RNA component, is the most important unit of the telomerase complex. The TERT gene promoter region has SP1-, ETS2-, and MYC-binding sites, and the three could synergistically activate TERT transcription and direct telomere elongation [46]. Among them, SP1 is effective but not necessary. SP1 overexpression can activate TERT, but SP1 site mutation has little effect on the TERT [47] activity. ETS2 is important for driving *TERT* gene expression, and silencing of ETS2 results in decreased *TERT* gene expression [48]. MYC plays an important role in TERT expression and telomere elongation. MYC regulates TERT in dual directions, with feed-forward regulation [49]. MYC can activate telomerase to extend the terminal telomere of the gene and return the cell to a sustained division state [50]. From our results, SP1, ETS2, and MYC all belong to the GDF11 transcriptomic network. Transcription of *TERT* can be inhibited by E2F transcription factor 1 (E2F1) [51], which is also involved in the GDF11-related transcription factor regulatory network. These results strongly suggest that GDF11 may have bidirectional regulation of *TERT* and telomere length. It can promote telomere synthesis by upregulating *TERT* transcription through SP1, ETS2, and MYC. At the same time, TERT and telomere synthesis can be inhibited by MYC and E2F1, and the MYC feed-forward reaction can precisely regulate TERT levels. This bidirectional regulation of telomerase is necessary. TERT overexpression induces tumor-like hyperproliferation, which is unfavorable for tissue and organ stability. However, with the bidirectional regulation of *TERT* transcription, telomeres can be regulated by controlling levels, which prolong telomere length, slow down cell senescence, restore organ function, maintain body homeostasis, and prolong the life of an organism to a certain extent. A recent study has shown that GDF11 has a positive effect on the maintenance of telomere length, which supports the conclusions of this study; however, it does not provide a specific mechanistic analysis [52]. In this study, bioinformatics analyses showed that GDF11 could operate through the SMAD/CTCF/MYC signaling pathway, SP1, and ETS2 to regulate *TERT* transcription to maintain telomere length, which provides new directions and targets for subsequent experimental verification.

## 5 Conclusion

From our bioinformatics network analyses, it could be concluded that inhibiting apoptosis, repairing DNA damage, and maintaining telomere length are closely related to aging. These are the main mechanisms for promoting proliferation and maintaining normal physiological functions in aging organisms, which can explain the anti-aging effects of GDF11 in various tissues and organs. The results of our gene co-expression network and transcription factor network analyses complemented each other. Taken together, in our study, these functions of GDF11 could explain the anti-aging phenomenon and involve the related transcription factors in the process, including MYC, SP1, and ETS2.

In summary, our comprehensive network analysis of GDF11 provides *in silico* predictions of GDF11 functions, revealing both previously known (such as transcriptional regulation) and novel (such as regulation of apoptosis, DNA repair, and telomerase) functions of GDF11, which may underlie its anti-aging effects. Although we used multiple credible databases and complementary methodologies to cross-validate our findings, the accuracy of our results relies on the quality and comprehensiveness of the datasets involved, and further experimental testing is warranted to validate our predictions resulting from network analyses. It is our goal that these findings contribute to clarifying mechanisms involving GDF11 in rejuvenation and identify candidate biomarkers or therapeutic targets for future research.

## Supplementary Material

Supplementary Figure
