# Supplementary material for: Bioinformatics network analyses of growth differentiation factor 11
Source: Open Life Sci. 2022 Apr 26;17(1):426–37. doi: 10.1515/biol-2022-0044 (PMC9055169; doi:10.1515/biol-2022-0044)
Supplement: Supplementary Figure [file biol-2022-0044-sm.pdf]

Supplementary Information

Bioinformatic network analyses of growth differentiation factor 11

Feng Zhang [1, 2, 3, 4, 5], Xia Yang [4] *, Zhijun Bao [1, 2, 3] *.

[1] Huadong Hospital Affiliated to Fudan University, 221 West Yan an Road, Shanghai, 200040, China.
[2] National Clinical Research Center for Aging and Medicine, Huashan Hospital, Fudan University, 12 Mid Urumqi Road, Shanghai, 200040, China.
[3] Shanghai Key Laboratory of Clinical Geriatric Medicine, 221 West Yan an Road, Shanghai, 200040, China.
[4] Department of Integrative Biology and Physiology, University of California, Los Angeles, 610 Charles E. Young Dr. E, Terasaki Life Sciences Bldg. Rm 2000B, Los Angeles, CA90095, USA.
[5] Department of Geriatrics, Huashan Hospital Affiliated to Fudan University, 12 Mid Urumqi Road, Shanghai, 200040, China.
* These authors contributed equally to this work.

Corresponding author:
Zhijun Bao
221 West Yan An Road, Shanghai, China, 200040
telephone and fax: (+86)021-62495877
e-mail address: xinyi8681@sina.com

Supplementary Table S1: GDF11 Genetic Co-expression Module Number of Each Human Tissue

Tissue		Module Amount
**Adipose**	Visceral omentum	1
**Nervous System**	Cerebellum	1
	Cerebellar hemisphere	1
	Frontal cortex	1
	Hippocampus	1
	Nerve tibialis	1

**Cardiovascular System**	Left ventricle	1
**Digestive System**	Esophagus mucosa	1
	Esophagus muscle	1
	Stomach	1
	Small intestine terminal ileum	1
	Colon transverse	1
	Colon sigmoid	1
**Liver**		1
**Skeletal Muscle**		1
**Lung**		1
**Kidney**		1
**Endocrine System**	Pituitary	1
	Thyroid	1
	Adrenal gland	1
**Female Reproduction System**	Ovary	1
**Male Reproduction System**	Testis	1
	Prostate	1
**Total**		23

Supplementary Table S2: Pathways and Functions of GDF11 Gene Co-expression Networks of Human

Tissue		Top pathways/functions	Overlaps	Genes belong to the pathway	Fold Enrichment	p-value	FDR q-value
Adipose	Visceral omentum	Genes involved in Generic Transcription Pathway (**Transcription**)	121	352	6.35	1.79 e-63	1.92 e-60
		DNA replication (**Proliferation**)	13	36	6.67	2.36 e-8	6.36 e-6
		Genes involved in Lagging Strand Synthesis (**Proliferation**)	9	19	8.75	2.21 e-7	2.86 e-5
		Genes involved in DNA strand elongation (**Proliferation**)	11	30	6.77	2.39 e-7	2.86 e-5
		Genes involved in Extension of Telomeres (**Telomere maintenance**)	10	27	6.84	7.63 e-7	8.21 e-5
		Genes involved in Global Genomic Nucleotide Excision Repair (**DNA Repair**)	11	35	5.80	1.42 e-6	1.39 e-4
		Genes involved in Processive synthesis on the lagging strand (**Proliferation**)	7	15	8.62	5.9 e-6	4.24 e-4
		Base excision repair (**DNA Repair**)	10	35	5.27	1.11 e-5	6.67 e-4
		Genes involved in Activation of the pre-replicative complex (**Proliferation**)	9	31	5.36	2.67 e-5	1.2 e-3
		Caspase Cascade in Apoptosis (**Apoptosis**)	7	23	5.62	1.53 e-4	4.46 e-3
		Mismatch repair (**DNA Repair**)	7	23	5.62	1.53 e-4	4.46 e-3

		Genes involved in Formation of incision complex in Global Genomic Nucleotide Excision Repair (**DNA Repair**)	7	23	5.62	1.53 e-4	4.46 e-3
		Genes involved in Homologous recombination repair of replication-independent double-strand breaks (**DNA Repair**)	6	17	6.52	1.84 e-4	4.79 e-3
		Genes involved in Inflammasomes (**Immune**)	6	17	6.52	1.84 e-4	4.79 e-3
		Genes involved in Cholesterol biosynthesis (**Metabolism of Cholesterol**)	7	24	5.38	2.06 e-4	4.93 e-3
		Genes involved in Double-Strand Break Repair (**DNA Repair**)	7	24	5.38	2.06 e-4	4.93 e-3
		Genes involved in The NLRP3 inflammasome (**Immune**)	5	12	7.69	2.66 e-4	6.09 e-3
		Genes involved in Base Excision Repair (**DNA Repair**)	6	19	5.83	3.67 e-4	7.61 e-3
		Genes involved in Base Excision Repair (**DNA Repair**)	6	19	5.83	3.67 e-4	7.61 e-3
		Genes involved in Polymerase switching (**Proliferation**)	5	13	7.10	4.12 e-4	8.08 e-3
Nervous System	Cerebellum	Genes involved in NCAM signaling for neurite out-growth (**NCAM signaling**)	5	64	39.89	1.79 e-7	1.93 e-4
		Genes involved in NCAM1 interactions (**NCAM signaling**)	4	39	52.37	1.07 e-6	5.3 e-4
		Extracellular matrix (ECM)-receptor	4	84	24.31	2.35 e-5	4.23 e-3

	interaction (**Extracellular matrix**)					
Cerebellar hemisphere	Multiple pathways from IGF-1R signaling lead to BAD phosphorylation (**IGF-1 signaling pathway**)	5	23	21.12	3.27 e-6	1.09 e-3
	IL-2 Receptor Beta Chain in T cell Activation (**Immune**)	5	38	12.78	4.29 e-5	3.35 e-3
	Genes involved in PI3K/AKT activation (**PI3K/AKT pathway**)	5	38	12.78	4.29 e-5	3.35 e-3
	IGF-1 Signaling Pathway (**IGF-1 signaling pathway**)	4	21	18.50	5.77 e-5	3.89 e-3
	Insulin Signaling Pathway (**Insulin pathway**)	4	22	17.66	7 e-5	4.43 e-3
	CTCF: First Multivalent Nuclear Factor (**Apoptosis**)	4	23	16.89	8.4 e-5	4.59 e-3
	Ras Signaling Pathway (**Ras pathway**)	4	23	16.89	8.4 e-5	4.59 e-3
	Double Stranded RNA Induced Gene Expression (**Immune**)	3	10	29.14	1.23 e-4	5.44 e-3
	Influence of Ras and Rho proteins on G1 to S Transition (**Cell cycle**)	4	26	14.94	1.38 e-4	5.44 e-3
	Phospholipids as signalling intermediaries (**Cell survival**)	4	27	14.39	1.61 e-4	5.79 e-3
	Inactivation of Gsk3 by AKT causes accumulation of b-catenin in Alveolar Macrophages (**AKT pathway**)	4	27	14.39	1.61 e-4	5.79 e-3
	Growth Hormone Signaling Pathway (**Growth**)	4	28	13.87	1.87 e-4	6.28 e-3
	Role of fl-arrestins in the activation and targeting of	3	12	24.28	2.22 e-4	7.05 e-3

	MAP kinases (**MAPK pathway**)					
	EGF Signaling Pathway (**EGF signaling**)	4	31	12.53	2.8 e-4	8.13 e-3
	Circadian rhythm – mammal (**Circadian rhythm**)	3	13	22.42	2.87 e-4	8.13 e-3
Frontal cortex	Arginine and proline metabolism (**Metabolism of amino acid**)	11	54	11.47	2.47 e-9	1.33 e-6
	Alanine, aspartate and glutamate metabolism (**Metabolism of amino acid**)	8	32	14.07	6.89 e-8	1.48 e-5
	Genes involved in tRNA Aminoacylation (**Expression**)	8	42	10.72	6.61 e-7	8.9 e-5
	Genes involved in Cytosolic tRNA aminoacylation (**Expression**)	6	24	14.07	3.15 e-6	3.77 e-4
	Aminoacyl-tRNA biosynthesis (**Expression**)	7	41	9.61	7.21 e-6	6.36 e-4
	Genes involved in Chondroitin sulfate biosynthesis (**Metabolism of GAG**)	5	21	13.40	2.8 e-5	1.89 e-3
	Proximal tubule bicarbonate reclamation (**Metabolism of ion**)	5	23	12.24	4.5 e-5	2.85 e-3
	Genes involved in Inhibition of voltage gated Ca2+ channels via G beta/gamma subunits (**Ca2+ pathway**)	5	25	11.26	6.9 e-5	4.13 e-3
	Genes involved in Amino acid synthesis and interconversion (**Metabolism of amino acid**)	4	17	13.25	1.95 e-4	8.06 e-3
	Genes involved in Rap1 signaling (**Rap1 signaling**)	4	17	13.25	1.95 e-4	8.06 e-3

Hippocampus	Genes involved in Metabolism of nucleotides (**Metabolism of nucleotides**)	8	72	6.78	2.38 e-5	3.6 e-3
	Sphingolipid metabolism (**Metabolism of sphingolipid**)	6	40	9.15	4.53 e-5	4.88 e-3
	Genes involved in Apoptotic cleavage of cellular proteins (**Apoptosis**)	6	40	9.15	4.53 e-5	4.88 e-3
	Axon guidance (**Axon guidance**)	10	129	4.73	5.62 e-5	5.5 e-3
Nerve tibia	Genes involved in ER-Phagosome pathway (**Phagocytosis**)	7	61	202.83	3.28 e-15	3.53 e-12
	Genes involved in Antigen processing-Cross presentation (**Immune**)	7	76	162.79	1.63 e-14	5.86 e-12
	Antigen processing and presentation (**Immune**)	6	89	119.15	9.92 e-12	1.23 e-9
	Genes involved in Endosomal/Vacuolar pathway (**Phagocytosis**)	4	9	785.57	1.01 e-11	1.23 e-9
	Genes involved in Interferon alpha/beta signaling (**Immune**)	5	64	138.08	2.87 e-10	3.09 e-8
	Genes involved in Antigen Presentation: Folding, assembly and peptide loading of class I MHC (**Immune**)	4	21	336.67	4.78 e-10	4.68 e-8
	Genes involved in Interferon gamma signaling (**Immune**)	4	63	112.22	4.69 e-8	3.88 e-6
	Genes involved in Immunoregulatory interactions between a Lymphoid and a non-Lymphoid cell (**Immune**)	4	70	101.00	7.19 e-8	5.53 e-6
	Allograft rejection (**Immune**)	3	38	139.54	1.34 e-6	8.48 e-5

	Graft-versus-host disease (**Immune**)	3	42	126.25	1.82 e-6	1.09 e-4
	Type I diabetes mellitus (**Immune**)	3	44	120.51	2.1 e-6	1.19 e-4
	Proteasome (**Proteasome pathway**)	3	48	110.47	2.73 e-6	1.34 e-4
	Genes involved in CDK-mediated phosphorylation and removal of Cdc6 (**DNA damage checkpoint**)	3	48	110.47	2.73 e-6	1.34 e-4
	Genes involved in Cross-presentation of soluble exogenous antigens (**Immune**)	3	48	110.47	2.73 e-6	1.34 e-4
	Genes involved in Regulation of ornithine decarboxylase (ODC) (**Metabolism of amino acid**)	3	49	108.21	2.91 e-6	1.36 e-4
	Genes involved in Autodegradation of the E3 ubiquitin ligase COP1 (**DNA damage checkpoint**)	3	51	103.97	3.29 e-6	1.36 e-4
	Genes involved in p53-Independent G1/S DNA damage checkpoint (**DNA damage checkpoint**)	3	51	103.97	3.29 e-6	1.36 e-4
	Genes involved in SCF-beta-TrCP mediated degradation of Emi1 (**DNA damage checkpoint**)	3	51	103.97	3.29 e-6	1.36 e-4
	Genes involved in Vif-mediated degradation of APOBEC3G (**Proteasome pathway**)	3	52	101.97	3.49 e-6	1.37 e-4
	Autoimmune thyroid disease (**Immune**)	3	53	100.04	3.7 e-6	1.37 e-4
	Genes involved in Destabilization of mRNA	3	53	100.04	3.7 e-6	1.37 e-4

		by AUF1 (hnRNP D0) (**Metabolism of GDP**)					
Cardiovascular System	Left ventricle	Genes involved in G alpha (12/13) signalling events (**GPCR pathway**)	9	74	9.77	3.63 e-7	9.77 e-5
		Aminoacyl-tRNA biosynthesis (**Expression**)	7	41	13.71	6.95 e-7	1.1 e-4
		Genes involved in NRAGE signals death through JNK (**Apoptosis**)	7	43	13.07	9.75 e-7	1.17 e-4
		Genes involved in Cell death signalling via NRAGE, NRIF and NADE (**Apoptosis**)	7	60	9.37	9.74 e-6	6.14 e-4
		Genes involved in trans-Golgi Network Vesicle Budding (**Vesicle-mediated transport**)	7	60	9.37	9.74 e-6	6.14 e-4
		Genes involved in Double-Strand Break Repair (**DNA repair**)	5	24	16.73	1.03 e-5	6.14 e-4
		Genes involved in tRNA Aminoacylation (**Expression**)	6	42	11.47	1.3 e-5	6.99 e-4
		Glycerolipid metabolism (**Metabolism of glycerolipid**)	6	49	9.83	3.22 e-5	1.24 e-3
		Genes involved in Metabolism of non-coding RNA (**Metabolism of non-coding RNA**)	6	49	9.83	3.22 e-5	1.24 e-3
		Other glycan degradation (**Metabolism of glycan**)	4	16	20.08	3.84 e-5	1.38 e-3
		Genes involved in Homologous recombination repair of replication-independent double-strand breaks (**DNA repair**)	4	17	18.90	4.97 e-5	1.65 e-3
		Genes involved in RNA Polymerase III Transcription (**Expression**)	5	33	12.17	5.22 e-5	1.65 e-3

	Telomeres, Telomerase, Cellular Aging, and Immortality (**Telomere maintenance**)	4	18	17.85	6.33 e-5	1.84 e-3
	Thrombin signaling and protease-activated receptors (**Thrombin signaling**)	5	37	10.85	9.21 e-5	2.36 e-3
	Genes involved in Mitochondrial tRNA aminoacylation (**Expression**)	4	21	15.30	1.2 e-4	2.94 e-3
	Genes involved in Lysosome Vesicle Biogenesis (**Vesicle-mediated transport**)	4	23	13.97	1.74 e-4	3.84 e-3
	Regulation of eIF4e and p70 S6 Kinase (**Expression**)	4	24	13.39	2.07 e-4	4.21 e-3
	Genes involved in Synthesis of PIPs at the late endosome membrane (**Metabolism of phospholipids**)	3	10	24.10	2.16 e-4	4.3 e-3
	Regulation of BAD phosphorylation (**Apoptosis**)	4	26	12.36	2.86 e-4	5.46 e-3
	Phospholipids as signalling intermediaries (**Metabolism of phospholipids**)	4	27	11.90	3.32 e-4	5.46 e-3
	Inactivation of Gsk3 by AKT causes accumulation of b-catenin in Alveolar Macrophages (**Proteosome pathway**)	4	27	11.90	3.32 e-4	5.46 e-3
	Genes involved in Synthesis of PIPs at the early endosome membrane (**Metabolism of phospholipids**)	3	12	20.08	3.88 e-4	6.24 e-3
	EGF Signaling Pathway (**EGF pathway**)	4	31	10.36	5.72 e-4	8.81 e-3

		PKC-catalyzed phosphorylation of inhibitory phosphoprotein of myosin phosphatase (**Smooth muscle contraction**)	4	31	10.36	5.72 e-4	8.81 e-3
		Non-homologous end-joining (**DNA repair**)	3	14	17.21	6.3 e-4	9.56 e-3
		PDGF Signaling Pathway (**PDGF pathway**)	4	32	10.04	6.48 e-4	9.56 e-3
Digestive System	Esophagus mucosa	Genes involved in Regulation of Complement cascade (**Immune**)	3	14	83.45	5.88 e-6	2.11 e-3
		Glycosaminoglycan biosynthesis - chondroitin sulfate (**Metabolism of GAG**)	3	22	53.10	2.45 e-5	6.6 e-3
	Esophagus muscle	Genes involved in Unfolded Protein Response (**ER stress**)	9	80	94.00	5.49 e-16	4.12 e-13
		Genes involved in Activation of Chaperone Genes by XBP1(S) (**ER stress**)	5	46	90.82	2.69 e-9	7.24 e-7
		Protein export (**Metabolism of protein**)	4	24	139.26	1.92 e-8	4.13 e-6
		Genes involved in Activation of Chaperone Genes by ATF6-alpha (**ER stress**)	3	11	227.88	2.66 e-7	4.09 e-5
		Genes involved in Activation of Chaperones by ATF6-alpha (**ER stress**)	3	13	192.82	4.6 e-7	6.19 e-5
		Genes involved in Antigen Presentation: Folding, assembly and peptide loading of class I MHC (**Metabolism of protein**)	3	21	119.36	2.12 e-6	2.16 e-4
	Stomach	Genes involved in Extracellular matrix	17	87	6.55	7.19 e-10	7.74 e-7

	organization (**Extracellular matrix**)					
	Genes involved in Collagen formation (**Collagen formation**)	13	58	7.52	1.25 e-8	3.36 e-6
	Neuropeptides VIP and PACAP inhibit the apoptosis of activated T cells (**Immune**)	8	29	9.26	1.49 e-6	2.01 e-4
	Genes involved in Response to elevated platelet cytosolic Ca2+ (**DAG/IP3/Ca2+ pathway**)	13	89	4.90	2.37 e-6	2.83 e-4
	Amino sugar and nucleotide sugar metabolism (**Metabolism of amino sugar and nucleotide sugar**)	9	44	6.86	4.98 e-6	4.47 e-4
	Phosphatidylinositol signaling system (**PI signaling**)	11	76	4.85	1.53 e-5	9.87 e-4
	VEGF signaling pathway (**PI signaling**)	11	76	4.85	1.53 e-5	9.87 e-4
	Genes involved in Semaphorin interactions (**Axon guidance**)	10	68	4.93	3.22 e-5	1.8 e-3
	Genes involved in CRMPs in Sema3A signaling (**Axon guidance**)	5	14	11.98	3.72 e-5	1.91 e-3
	Genes involved in Glutamate Neurotransmitter Release Cycle (**Neurotransmitter release cycle**)	5	15	11.18	5.45 e-5	2.55 e-3
	Signaling Pathway from G-Protein Families (**DAG/IP3/Ca2+ pathway**)	7	37	6.35	9.65 e-5	4 e-3
	Basal cell carcinoma (**Hedgehog signaling**)	8	55	4.88	2.13 e-4	6.56 e-3
	Effects of calcineurin in Keratinocyte	5	21	7.99	3.18 e-4	8.61 e-3

	Differentiation (**DAG/IP3/Ca2+ pathway**)					
	Phospholipase C-epsilon pathway (**DAG/IP3/Ca2+ pathway**)	4	12	11.18	3.2 e-4	8.61 e-3
Small intestine terminal ileum	Genes involved in Biological oxidations (**Biological oxidation**)	31	139	7.51	1.26 e-18	4.54 e-16
	Genes involved in Phase II conjugation (**Biological oxidation**)	17	70	8.18	1.72 e-11	2.32 e-9
	Genes involved in Glycerophospholipid biosynthesis (**Metabolism of glycerophospholipid**)	17	82	6.99	2.53 e-10	3.03 e-8
	Retinol metabolism (**Metabolism of retinol**)	15	64	7.90	4.57 e-10	4.48 e-8
	Genes involved in Lipid digestion, mobilization, and transport (**Metabolism of lipids**)	13	46	9.52	5.32 e-10	4.77 e-8
	PPAR signaling pathway (**PPAR signaling pathway**)	15	69	7.32	1.41 e-9	1.17 e-7
	Drug metabolism - other enzymes (**Metabolism of drug**)	12	51	7.93	2.41 e-8	1.23 e-6
	Genes involved in Cytochrome P450 - arranged by substrate type (**Biological oxidation**)	12	51	7.93	2.41 e-8	1.23 e-6
	Genes involved in Lipoprotein metabolism (**Metabolism of lipids**)	9	28	10.83	7.18 e-8	3.22 e-6
	Genes involved in Amino acid and oligopeptide SLC transporters (**Metabolism of amino acid**)	11	49	7.56	1.55 e-7	5.58 e-6
	Genes involved in Nuclear Receptor transcription pathway (**Transcription**)	11	49	7.56	1.55 e-7	5.58 e-6

	Sphingolipid metabolism (**Metabolism of sphingolipid**)	10	40	8.42	1.92 e-7	6.67 e-6
	Genes involved in Gluconeogenesis (**Metabolism of gluconeogenesis**)	9	34	8.92	4.65 e-7	1.52 e-5
	Genes involved in Glycosphingolipid metabolism (**Metabolism of glycosphingolipid**)	9	38	7.98	1.3 e-6	3.68 e-5
	Genes involved in Transport of vitamins, nucleosides, and related molecules (**Membrane transport**)	8	31	8.70	2.52 e-6	6.63 e-5
	Genes involved in Xenobiotics (**Biological oxidation**)	6	16	12.64	4.18 e-6	1.02 e-4
	Renin-angiotensin system (**Renin-angiotensin system**)	6	17	11.90	6.29 e-6	1.51 e-4
	Genes involved in Triglyceride Biosynthesis (**Metabolism of lipids**)	8	38	7.09	1.3 e-5	2.91 e-4
	Linoleic acid metabolism (**Metabolism of linoleic acid**)	7	29	8.13	1.75 e-5	3.7 e-4
	Genes involved in Glycolysis (**Metabolism of glycolysis**)	7	29	8.13	1.75 e-5	3.7 e-4
	Genes involved in ERK/MAPK targets (**ERK/MAPK pathway**)	6	21	9.63	2.49 e-5	5.06 e-4
	Genes involved in Amino acid transport across the plasma membrane (**Membrane transport**)	7	31	7.61	2.8 e-5	5.43 e-4
	Nuclear Receptors in Lipid Metabolism and Toxicity (**Metabolism of lipids and toxin)**	5	15	11.23	5.34 e-5	8.98 e-4

	Genes involved in HDL-mediated lipid transport (**Metabolism of lipids**)	5	15	11.23	5.34 e-5	8.98 e-4
	Genes involved in Nuclear Events (**Transcription**)	6	24	8.42	5.73 e-5	9.49 e-4
	Genes involved in Chylomicron-mediated lipid transport (**Metabolism of lipids**)	5	16	10.53	7.57 e-5	1.2 e-3
	Genes involved in Recycling pathway of L1 (**Axon guidance**)	6	27	7.49	1.17 e-4	1.7 e-3
	Genes involved in Synthesis of PA (**Metabolism of phospholipids**)	6	27	7.49	1.17 e-4	1.7 e-3
	Pentose and glucuronate interconversions (**Metabolism of glucose**)	6	28	7.22	1.45 e-4	2.03 e-3
	Genes involved in Peroxisomal lipid metabolism (**Metabolism of lipids**)	5	21	8.02	3.12 e-4	3.65 e-3
	Cystic Fibrosis Transmembrane Conductance Regulator And Beta 2 Adrenergic Receptor Pathway (**cAMP related signaling**)	4	12	11.23	3.15 e-4	3.65 e-3
	Genes involved in Apoptotic cleavage of cell adhesion proteins (**Apoptosis**)	4	12	11.23	3.15 e-4	3.65 e-3
	Genes involved in ERKs are inactivated (**ERK/MAPK pathway**)	4	12	11.23	3.15 e-4	3.65 e-3
	Genes involved in Metal ion SLC transporters (**Membrane transport**)	5	22	7.66	3.94 e-4	4.24 e-3
Colon transverse	Extracellular matrix (ECM)-receptor interaction (**Extracellular matrix**)	10	84	6.40	3.72 e-6	5.73 e-4

	Genes involved in Sphingolipid metabolism (**Metabolism of phospholipids**)	9	69	7.01	5.3 e-6	7.14 e-4
	Fructose and mannose metabolism (**Metabolism of glycose**)	6	34	9.49	3.49 e-5	2.92 e-3
	Genes involved in Synthesis of PE (**Metabolism of phospholipids**)	4	11	19.56	3.52 e-5	2.92 e-3
	Genes involved in NCAM1 interactions (**NCAM signaling**)	6	39	8.27	7.82 e-5	4.6 e-3
	Genes involved in Effects of PIP2 hydrolysis (**Metabolism of phospholipids**)	5	25	10.76	8.54 e-5	4.6 e-3
	Mechanism of Gene Regulation by Peroxisome Proliferators via PPARa (**Transcription regulation**)	7	58	6.49	9.85 e-5	4.82 e-3
	Genes involved in Collagen formation (**Collagen formation**)	7	58	6.49	9.85 e-5	4.82 e-3
	Genes involved in Regulation of Insulin Secretion by Glucagon-like Peptide-1 (**GLP-1 and insulin secretion**)	6	43	7.50	1.37 e-4	5.91 e-3
Colon sigmoid	Genes involved in Cytosolic tRNA aminoacylation (**Expression**)	8	24	62.27	4.11 e-13	2.21 e-10
	Genes involved in tRNA Aminoacylation (**Expression**)	9	42	40.03	1.19 e-12	4.28 e-10
	Aminoacyl-tRNA biosynthesis (**Expression**)	7	41	31.89	2.23 e-9	5.99 e-7
	Genes involved in Metabolism of non-coding	7	49	26.68	8.2 e-9	1.77 e-6

	RNA (**Metabolism of non-coding RNA**)					
	Genes involved in Protein folding (**Expression**)	7	53	24.67	1.44 e-8	2.59 e-6
	Genes involved in Prefoldin mediated transfer of substrate to CCT/TriC (**Expression**)	5	28	33.35	3.75 e-7	4.04 e-5
	Glutathione metabolism (**Metabolism of glutathione**)	5	50	18.68	7.34 e-6	4.65 e-4
	Genes involved in Association of TriC/CCT with target proteins during biosynthesis (**Expression**)	4	27	27.67	1.28 e-5	6.87 e-4
	Genes involved in NEP/NS2 Interacts with the Cellular Export Machinery (**Expression**)	4	27	27.67	1.28 e-5	6.87 e-4
	Genes involved in Purine ribonucleoside monophosphate biosynthesis (**Metabolism of purine ribonucleoside**)	3	11	50.94	2.42 e-5	1.19 e-3
	One carbon pool by folate (**Biological oxidation**)	3	17	32.96	9.75 e-5	3.89 e-3
	Genes involved in Formation of tubulin folding intermediates by CCT/TriC (**Expression**)	3	22	25.47	2.16 e-4	7.06 e-3
	Genes involved in RNA Polymerase I Transcription Termination (**Expression**)	3	22	25.47	2.16 e-4	7.06 e-3
	Genes involved in RNA Polymerase I Transcription Initiation (**Expression**)	3	25	22.41	3.19 e-4	9.05 e-3
Liver	Genes involved in Extracellular matrix organization (**Extracellular matrix**)	17	87	21.38	4.32 e-18	4.65 e-15

	Genes involved in Signaling by PDGF (**PDGF signaling**)	18	122	16.14	8.65 e-17	4.66 e-14
	Genes involved in Collagen formation (**Collagen formation**)	14	58	26.41	1.61 e-16	5.77 e-14
	Extracellular matrix (ECM)-receptor interaction (**Extracellular matrix**)	13	84	16.93	8.97 e-13	1.38 e-10
	Genes involved in NCAM1 interactions (**NCAM signaling**)	9	39	25.25	6.79 e-11	8.13 e-9
	Genes involved in NCAM signaling for neurite out-growth (**NCAM signaling**)	10	64	17.09	3.57 e-10	3.84 e-8
	Genes involved in Muscle contraction (**Muscle contraction**)	7	48	15.95	2.7 e-7	1.94 e-5
	Genes involved in Chondroitin sulfate/dermatan sulfate (**metabolism Metabolism of GAG**)	7	49	15.63	3.13 e-7	2.1 e-5
	Genes involved in Smooth Muscle Contraction (**Smooth Muscle Contraction**)	5	25	21.88	2.85 e-6	1.7 e-4
	PKC-catalyzed phosphorylation of inhibitory phosphoprotein of myosin phosphatase (**Smooth muscle contraction**)	5	31	17.64	8.7 e-6	4.46 e-4
	Role of EGF Receptor Transactivation by GPCRs in Cardiac Hypertrophy (**GRCP signaling pathway**)	4	18	24.31	1.9 e-5	8.67 e-4
	Genes involved in Chondroitin sulfate biosynthesis (**Metabolism of GAG**)	4	21	20.84	3.64 e-5	1.22 e-3

	Genes involved in A tetrasaccharide linker sequence is required for GAG synthesis (**Metabolism of GAG**)	4	25	17.50	7.47 e-5	2.3 e-3
	Genes involved in Keratan sulfate degradation (**Metabolism of GAG**)	3	11	29.84	1.18 e-4	3.54 e-3
	Genes involved in Receptor-ligand binding initiates the second proteolytic cleavage of Notch receptor (**NOTCH pathway**)	3	12	27.35	1.57 e-4	4.22 e-3
	Genes involved in Signaling by NOTCH3 (**NOTCH pathway**)	3	12	27.35	1.57 e-4	4.22 e-3
Skeletal Muscle	Genes involved in Fatty acid, triacylglycerol, and ketone body metabolism (**Metabolism of lipids**)	29	168	6.10	6.2 e-15	6.68 e-12
	HIV-I Nef: negative effector of Fas and TNF (**Immune**)	14	58	8.53	6.16 e-10	1.58 e-7
	Genes involved in Muscle contraction (**Muscle contraction**)	12	48	8.84	6.73 e-9	1.04 e-6
	Genes involved in Striated Muscle Contraction (**Muscle contraction**)	9	27	11.79	3.32 e-8	3.97 e-6
	Genes involved in TRIF mediated TLR3 signaling (**Toll-like receptor pathway**)	13	74	6.21	1.46 e-7	9.18 e-6
	Genes involved in NFkB and MAP kinases activation mediated by TLR4 signaling repertoire (**Toll-like receptor pathway, NF-κB pathway and MAPK pathway**)	12	72	5.89	7.95 e-7	3.43 e-5
	Genes involved in Transcriptional Regulation of White Adipocyte	12	72	5.89	7.95 e-7	3.43 e-5

	Differentiation (**Transcription regulation**)					
	Induction of apoptosis through DR3 and DR4/5 Death Receptors (**Apoptosis**)	8	33	8.57	2.95 e-6	9.63 e-5
	Genes involved in Activation of Chaperone Genes by XBP1(S) (**ER stress**)	9	46	6.92	4.81 e-6	1.4 e-4
	Genes involved in MAP kinase activation in TLR cascade (**Toll-like receptor pathway**)	9	50	6.36	9.89 e-6	2.6 e-4
	FAS signaling pathway (CD95) (**Apoptosis**)	7	30	8.25	1.63 e-5	3.91 e-4
	Inositol phosphate metabolism (**Metabolism of phospholipids**)	9	54	5.89	1.9 e-5	4.35 e-4
	Apoptotic Signaling in Response to DNA Damage (**Apoptosis**)	6	22	9.64	2.55 e-5	5.32 e-4
	Rho cell motility signaling pathway (**Muscle contraction**)	7	32	7.73	2.57 e-5	5.32 e-4
	Alanine, aspartate and glutamate metabolism (**Metabolism of amino acid**)	7	32	7.73	2.57 e-5	5.32 e-4
	Valine, leucine and isoleucine degradation (**Metabolism of amino acid**)	8	44	6.43	2.86 e-5	5.72 e-4
	N-Glycan biosynthesis (**Metabolism of glycoprotein**)	8	46	6.15	4.01 e-5	7.31 e-4
	Genes involved in RORA Activates Circadian Expression (**Circadian rhythm**)	6	24	8.84	4.38 e-5	7.74 e-4

	SNARE interactions in vesicular transport (**Vesicular transport**)	7	38	6.51	8.31 e-5	1.36 e-3
	Genes involved in Triglyceride Biosynthesis (**Metabolism of lipids**)	7	38	6.51	8.31 e-5	1.36 e-3
	Genes involved in Branched-chain amino acid catabolism (**Metabolism of amino acid**)	5	17	10.40	8.34 e-5	1.36 e-3
	Fatty acid metabolism (**Metabolism of lipids**)	7	42	5.89	1.61 e-4	2.38 e-3
	Genes involved in Intrinsic Pathway for Apoptosis (**Apoptosis**)	6	30	7.07	1.67 e-4	2.43 e-3
	Genes involved in ERK/MAPK targets (**ERK/MAPK pathway**)	5	21	8.42	2.5 e-4	3.4 e-3
	Genes involved in ERKs are inactivated (**ERK/MAPK pathway**)	4	12	11.79	2.62 e-4	3.53 e-3
	Signal transduction through IL1R (**NF-κB pathway and MAPK pathway**)	6	33	6.43	2.9 e-4	3.83 e-3
	Genes involved in Gluconeogenesis (**Metabolism of glucose**)	6	34	6.24	3.44 e-4	4.41 e-3
	Circadian rhythm – mammal (**Circadian rhythm**)	4	13	10.88	3.7 e-4	4.64 e-3
	Genes involved in Circadian Repression of Expression by REV-ERBA (**Circadian rhythm**)	5	23	7.69	3.94 e-4	4.88 e-3
	Genes involved in BMAL1:CLOCK/NPAS2 Activates Circadian Expression (**Circadian rhythm**)	6	36	5.89	4.75 e-4	5.69 e-3

		Nicotinate and nicotinamide metabolism (**Metabolism of vitamin**)	5	24	7.37	4.86 e-4	5.69 e-3
		Genes involved in Nuclear Events (kinase and transcription factor activation) (**Transcription**)	5	24	7.37	4.86 e-4	5.69 e-3
		Cell to Cell Adhesion Signaling (**Cellular activities**)	4	14	10.10	5.07 e-4	5.87 e-3
		Genes involved in Smooth Muscle Contraction (**Muscle contraction**)	5	25	7.07	5.94 e-4	6.66 e-3
Lung		Genes involved in Ion transport by P-type ATPases (**Ion transport**)	5	34	17.32	9.75 e-6	1.68 e-3
		Genes involved in Acyl chain remodelling of phosphatidylcholine (**Metabolism of phospholipids**)	4	22	21.42	3.31 e-5	3.56 e-3
		Genes involved in Metabolism of vitamins and cofactors (**Metabolism of vitamin**)	5	51	11.55	7.31 e-5	6.56 e-3
		Genes involved in Ion channel transport (**Ion transport**)	5	55	10.71	1.05 e-4	8.1 e-3
		Genes involved in HS-GAG biosynthesis (**Metabolism of GAG**)	4	31	15.20	1.34 e-4	7.21 e-3
Kidney		Extracellular matrix (ECM)-receptor interaction (**Extracellular matrix**)	8	31	18.38	2.46 e-5	8.84 e-3
Endocrine System	Thyroid	Genes involved in Generic Transcription Pathway (**Transcription**)	20	352	9.46	6.37 e-14	6.86 e-11
	Pituitary	Axon guidance (**Axon guidance**)	31	129	5.72	1.94 e-15	4.18 e-13

	Arginine and proline metabolism (**Metabolism of amino acid**)	18	54	7.93	3.49 e-12	4.18 e-10
	Fc gamma R-mediated phagocytosis (**Phagocytosis**)	23	97	5.64	1.07 e-11	1.15 e-9
	Extracellular matrix (ECM)-receptor interaction (**Extracellular matrix**)	21	84	5.95	2.83 e-11	2.54 e-9
	Genes involved in HDL-mediated lipid transport (**Metabolism of lipids**)	10	15	15.87	4.13 e-11	3.42 e-9
	Genes involved in Integrin cell surface interactions (**Extracellular matrix**)	18	79	5.42	3.65 e-9	1.87 e-7
	Genes involved in Glycerophospholipid biosynthesis (**Metabolism of phospholipids**)	18	82	5.22	6.88 e-9	3.37 e-7
	Genes involved in Lipoprotein metabolism (**Metabolism of lipids**)	11	28	9.35	7.72 e-9	3.62 e-7
	Arrhythmogenic right ventricular cardiomyopathy (ARVC) (**Ca2+ pathway**)	17	76	5.32	1.33 e-8	5.72 e-7
	Phosphoinositides and their downstream targets (**Metabolism of phospholipids**)	9	23	9.31	1.91 e-7	6.63 e-6
	Genes involved in Lipid digestion, mobilization, and transport (**Metabolism of lipids**)	12	46	6.21	2.99 e-7	1.01 e-5
	Genes involved in CRMPs in Sema3A signaling (**Axon guidance**)	7	14	11.90	6.03 e-7	1.8 e-5
	Genes involved in Collagen formation (**Collagen formation**)	13	58	5.33	6.54 e-7	1.9 e-5

	Genes involved in Netrin-1 signaling (**Axon guidanc**e)	11	41	6.38	6.86 e-7	1.9 e-5
	Glutathione metabolism (**Metabolism of glutathione**)	12	50	5.71	8 e-7	2.15 e-5
	Genes involved in Cell death signalling via NRAGE, NRIF and NADE (**Apoptosis**)	13	60	5.15	9.89 e-7	2.54 e-5
	Genes involved in NRAGE signals death through JNK (**Apoptosis**)	11	43	6.09	1.16 e-6	2.86 e-5
	Base excision repair (**DNA repair**)	10	35	6.80	1.17 e-6	2.86 e-5
	Endometrial cancer (**Cancer**)	12	52	5.49	1.26 e-6	2.95 e-5
	Cyclins and Cell Cycle Regulation (**Cell cycle**)	8	23	8.28	2.66 e-6	5.61 e-5
	Genes involved in Glutathione conjugation (**Metabolism of glutathione**)	8	23	8.28	2.66 e-6	5.61 e-5
	PKC-catalyzed phosphorylation of inhibitory phosphoprotein of myosin phosphatase (**Smooth muscle contraction**)	9	31	6.91	3.47 e-6	6.8 e-5
	Genes involved in Regulation of signaling by CBL (**Phagocytosis**)	7	18	9.25	4.82 e-6	8.95 e-5
	DNA replication (**Proliferation**)	9	36	5.95	1.34 e-5	1.99 e-4
	Genes involved in Myogenesis (**Myogenesis**)	8	28	6.80	1.4 e-5	2.03 e-4
	Keratinocyte Differentiation (**Differentiation**)	10	46	5.17	1.7 e-5	2.38 e-4
	Glycosaminoglycan biosynthesis - chondroitin	7	22	7.57	2.23 e-5	2.93 e-4

		sulfate (**Metabolism of GAG**)					
		O-Glycan biosynthesis (**Metabolism of glycan**)	8	30	6.34	2.44 e-5	3.09 e-4
		Proximal tubule bicarbonate reclamation (**Metabolism of ion**)	7	23	7.24	3.09 e-5	3.69 e-4
		Genes involved in Transport of vitamins, nucleosides, and related molecules (**Molecules transport**)	8	31	6.14	3.16 e-5	3.74 e-4
		Genes involved in Sulfur amino acid metabolism (**Metabolism of amino acid**)	7	24	6.94	4.2 e-5	4.92 e-4
		Genes involved in Dopamine Neurotransmitter Release Cycle (**Neurotransmitter Release**)	5	11	10.82	4.86 e-5	5.56 e-4
		Genes involved in Neurotransmitter Release Cycle (**Neurotransmitter Release**)	8	34	5.60	6.51 e-5	7.11 e-4
		Genes involved in Synthesis of phosphatidylcholine (**metabolism of phospholipids**)	6	18	7.93	6.53 e-5	7.11 e-4
	Adrenal gland	Genes involved in GAB1 signalosome **(PI3K/AKT pathway**)	7	38	13.69	6.76 e-7	2.74 e-4
		Genes involved in PI3K events in ERBB2 signaling (**PI3K/AKT pathway**)	6	44	10.14	2.64 e-5	2.84 e-3
		Genes involved in PIP3 activates AKT signaling (**PI3K/AKT pathway**)	5	29	12.82	3.93 e-5	3.85 e-3
		Genes involved in Nuclear Receptor transcription pathway (**Nuclear**	6	49	9.10	4.94 e-5	4.09 e-3

		**receptor transcription pathway**)					
		Fructose and mannose metabolism (**Metabolism of glucide**)	5	34	10.93	8.72 e-5	5.64 e-3
		Genes involved in PI3K/AKT activation (**PI3K/AKT pathway**)	5	38	9.78	1.5 e-4	7.72 e-3
		Genes involved in PI3K events in ERBB4 signaling (**PI3K/AKT pathway**)	5	38	9.78	1.5 e-4	7.72 e-3
Female Reproductive System	Ovary	Ribosome (**Expression**)	61	88	107.62	8.72 e-115	9.39 e-112
		Genes involved in 3' -UTR-mediated translational regulation (**Translational regulation**)	65	176	57.33	1.56 e-97	8.42 e-95
		Genes involved in Peptide chain elongation (**Expression**)	60	153	60.88	7.53 e-92	2.03 e-89
		Genes involved in Activation of the mRNA upon binding of the cap-binding complex and eIFs, and subsequent binding to 43S (**Expression**)	32	84	59.14	1.6 e-48	1.43 e-46
		Genes involved in Formation of the ternary complex, and subsequently, the 43S complex (**Expression**)	30	74	62.94	1.5 e-46	1.24 e-44
Male Reproductive System	Testis	Genes involved in Generic Transcription Pathway (**Transcription regulation**)	39	352	4.67	2.04 e-15	2.2 e-12
	Prostate	Regulation of BAD phosphorylation (**Apoptosis**)	4	26	28.05	1.2 e-5	1.18 e-3

	Genes involved in Keratan sulfate biosynthesis (**Metabolism of GAG**)	4	26	28.05	1.2 e-5	1.18 e-3
	Genes involved in Keratan sulfate/keratin metabolism (**Metabolism of GAG**)	4	30	24.31	2.16 e-5	1.79 e-3
	Genes involved in HS-GAG biosynthesis (**Metabolism of GAG**)	4	31	23.53	2.47 e-5	1.87 e-3
	Genes involved in Keratan sulfate degradation (**Metabolism of GAG**)	3	11	49.73	2.6 e-5	1.87 e-3
	Genes involved in Regulation of Insulin-like Growth Factor (IGF) Activity by Insulin-like Growth Factor Binding Proteins (IGFBPs) (**IGF signaling**)	3	16	34.19	8.65 e-5	5.48 e-3
	Genes involved in Regulation of KIT signaling (**Proliferation**)	3	17	32.18	1.05 e-4	5.93 e-3

Supplementary Table S3: Transcription Factor Network of GDF11 in Different Human Cells

System	Tissue	Amount	Network Member
Nervous System	Neural Stem Cell	22	SP3, RARG, KLF14, KLF12, KLF7, NFIC, CPEB1, SP8, CREB1, NFYA, IKZF2, MYF6, SP1, NFY, SP4, RFX3, RFX4, RFX1, KLF4, ATF1, RFX5, KLF16
	Neuron	8	CREB1, NFYA, NFY, ATF1, SP1, SP4, KLF16, IKZF2
	Brain	20	SP3, RARG, KLF14, KLF12, KLF7, NFIC, SP8, CREB1, NFYA, MYF6, SP1, NFY, SP4, RFX3, RFX4, RFX1, KLF4, ATF1, RFX5, KLF16
	Occipital Cortex	21	SP3, RARG, KLF14, KLF12, KLF7, NFIC, SP8, CREB1, NFYA, AP1, MYF6, SP1, NFY, SP4, RFX3, RFX4, RFX1, KLF4, ATF1, RFX5, KLF16
	Occipital Lobe	8	CREB1, NFYA, NFY, ATF1, SP1, SP4, KLF16, IKZF2
	Occipital Pole	20	SP3, RARG, KLF14, KLF12, KLF7, NFIC, SP8, CREB1, NFYA, MYF6, SP1, NFY, SP4, RFX3, RFX4, RFX1, KLF4, ATF1, RFX5, KLF16
	Paracentral Gyrus	10	CREB1, NFYA, NFY, ATF1, SP1, SP4, KLF16, RFX1, RFX4, RFX5
	Postcentral Gyrus	7	CREB1, NFYA, NFY, ATF1, SP1, SP4, KLF16
	Parietal Lobe	20	SP3, RARG, KLF14, KLF12, KLF7, NFIC, SP8, CREB1, NFYA, MYF6, SP1, NFY, SP4, RFX3, RFX4, RFX1, KLF4, ATF1, RFX5, KLF16
	Frontal Lobe	20	SP3, RARG, KLF14, KLF12, KLF7, NFIC, SP8, CREB1, NFYA, MYF6, SP1, NFY, SP4, RFX3, RFX4, RFX1, KLF4, ATF1, RFX5, KLF16
	Medial Frontal Gyrus	21	SP3, RARG, KLF14, KLF12, KLF7, NFIC, SP8, CREB1, NFYA, IKZF2, MYF6, SP1, NFY, SP4, RFX3, RFX4, RFX1, KLF4, ATF1, RFX5, KLF16

Medial Temporal Gyrus	20	SP3, RARG, KLF14, KLF12, KLF7, NFIC, SP8, CREB1, NFYA, MYF6, SP1, NFY, SP4, RFX3, RFX4, RFX1, KLF4, ATF1, RFX5, KLF16
Temporal Lobe	7	CREB1, NFYA, NFY, ATF1, SP1, SP4, KLF16
Hippocampus	21	SP3, RARG, KLF14, KLF12, KLF7, NFIC, SP8, CREB1, NFYA, IKZF2, MYF6, SP1, NFY, SP4, RFX3, RFX4, RFX1, KLF4, ATF1, RFX5, KLF16
Amygdala	20	SP3, RARG, KLF14, KLF12, KLF7, NFIC, SP8, CREB1, NFYA, MYF6, SP1, NFY, SP4, RFX3, RFX4, RFX1, KLF4, ATF1, RFX5, KLF16
Putamen	20	SP3, RARG, KLF14, KLF12, KLF7, NFIC, SP8, CREB1, NFYA, MYF6, SP1, NFY, SP4, RFX3, RFX4, RFX1, KLF4, ATF1, RFX5, KLF16
Caudate Nucleus	20	SP3, RARG, KLF14, KLF12, KLF7, NFIC, SP8, CREB1, NFYA, MYF6, SP1, NFY, SP4, RFX3, RFX4, RFX1, KLF4, ATF1, RFX5, KLF16
Corpus Callosum	7	CREB1, NFYA, NFY, ATF1, SP1, SP4, KLF16
Globus Pallidus	20	SP3, RARG, KLF14, KLF12, KLF7, NFIC, SP8, CREB1, NFYA, MYF6, SP1, NFY, SP4, RFX3, RFX4, RFX1, KLF4, ATF1, RFX5, KLF16
Nucleus Accumbens	20	SP3, RARG, KLF14, KLF12, KLF7, NFIC, SP8, CREB1, NFYA, MYF6, SP1, NFY, SP4, RFX3, RFX4, RFX1, KLF4, ATF1, RFX5, KLF16
Thalamus	4	CREB1, NFYA, NFY, ATF1
Pineal Gland	7	CREB1, NFYA, NFY, ATF1, SP1, SP4, KLF16
Substantia Nigra	5	CREB1, NFYA, NFY, ATF1, IKZF2
Pituitary	16	SP3, RARG, KLF14, KLF12, KLF7, NFIC, SP8, MYF6, SP1, SP4, RFX3, RFX4, RFX1, KLF4, RFX5, KLF16
Diencephalon	7	CREB1, NFYA, NFY, ATF1, SP1, SP4, KLF16
Pons	7	CREB1, NFYA, NFY, ATF1, SP1, SP4, KLF16
Medulla Oblongata	20	SP3, RARG, KLF14, KLF12, KLF7, NFIC, SP8, CREB1, NFYA, MYF6, SP1, NFY, SP4, RFX3, RFX4, RFX1, KLF4, ATF1, RFX5, KLF16
Cerebellum	21	SP3, RARG, KLF14, KLF12, KLF7, NFIC, SP8, CREB1, NFYA, IKZF2, MYF6, SP1, NFY, SP4, RFX3, RFX4, RFX1, KLF4, ATF1, RFX5, KLF16
Cerebral Meninges	7	CREB1, NFYA, NFY, ATF1, SP1, SP4, KLF16

	Spinal Cord	4	CREB1, NFYA, NFY, ATF1
	Olfactory Region	20	SP3, RARG, KLF14, KLF12, KLF7, NFIC, SP8, CREB1, NFYA, MYF6, SP1, NFY, SP4, RFX3, RFX4, RFX1, KLF4, ATF1, RFX5, KLF16
	Optic Nerve	7	CREB1, NFYA, NFY, ATF1, SP1, SP4, KLF16
	Retina	20	SP3, RARG, KLF14, KLF12, KLF7, NFIC, SP8, CREB1, NFYA, MYF6, SP1, NFY, SP4, RFX3, RFX4, RFX1, KLF4, ATF1, RFX5, KLF16
Cardiovascular System	Heart	4	CREB1, NFYA, NFY, ATF1
	Left Atrium	20	SP3, RARG, KLF14, KLF12, KLF7, NFIC, SP8, CREB1, NFYA, MYF6, SP1, NFY, SP4, RFX3, RFX4, RFX1, KLF4, ATF1, RFX5, KLF16
	Left Ventricle	4	CREB1, NFYA, NFY, ATF1
	Heart Mitral Valve	10	CREB1, NFYA, NFY, ATF1, SP1, SP4, KLF16, IKZF2, FEV, AP1
	Heart Pulmonic Valve	22	SP3, RARG, KLF14, KLF12, KLF7, NFIC, AP1, SP8, CREB1, NFYA, IKZF2, MYF6, SP1, NFY, SP4, RFX3, RFX4, RFX1, KLF4, ATF1, RFX5, KLF16
	Heart Tricuspid Valve	4	CREB1, NFYA, NFY, AP1
	Blood	4	CREB1, NFYA, NFY, ATF1
	Aorta	2	CREB1, NFYA
	Endothelial Cell Aorta	20	SP3, RARG, KLF14, KLF12, KLF7, NFIC, SP8, CREB1, NFYA, MYF6, SP1, NFY, SP4, RFX3, RFX4, RFX1, KLF4, ATF1, RFX5, KLF16
	Endothelial Cell Artery	7	CREB1, NFYA, NFY, ATF1, SP1, SP4, KLF16
	Endothelial Cell Microvascular	4	CREB1, NFYA, NFY, ATF1
	Vein	19	SP3, RARG, KLF14, KLF12, KLF7, NFIC, SP8, CREB1, NFYA, MYF6, SP1, NFY, SP4, RFX3, RFX4, RFX1, KLF4, RFX5, KLF16
	Pericyte	20	SP3, RARG, KLF14, KLF12, KLF7, NFIC, SP8, CREB1, NFYA, MYF6, SP1, NFY, SP4, RFX3, RFX4, RFX1, KLF4, ATF1, RFX5, KLF16
Digestive System	Hepatocyte	20	SP3, RARG, KLF14, KLF12, KLF7, NFIC, SP8, CREB1, NFYA, MYF6, SP1, NFY, SP4, RFX3, RFX4, RFX1, KLF4, ATF1, RFX5, KLF16

	Hepatic Sinusoid Endothelial Cell	7	CREB1, NFYA, NFY, ATF1, SP1, SP4, KLF16
	Hepatic Stellate Cell Lipocyte	7	CREB1, NFYA, NFY, ATF1, SP1, SP4, KLF16
	Gall Bladder	6	CREB1, NFYA, NFY, ATF1, SP1, SP4
	Insula	20	SP3, RARG, KLF14, KLF12, KLF7, NFIC, SP8, CREB1, NFYA, MYF6, SP1, NFY, SP4, RFX3, RFX4, RFX1, KLF4, ATF1, RFX5, KLF16
	Spleen	6	CREB1, NFYA, NFY, ATF1, SP1, SP4
	Appendix	7	CREB1, NFYA, NFY, ATF1, SP1, SP4, KLF16
	Small Intestine	2	CREB1, NFYA
Respiratory System	Throat	6	CREB1, NFYA, NFY, ATF1, SP1, SP4
	Trachea	3	CREB1, NFYA, NFY
Urinary system	Bladder	5	CREB1, NFYA, NFY, SP1, SP4
Skeletal Muscle	Osteoblast	20	SP3, RARG, KLF14, KLF12, KLF7, NFIC, SP8, CREB1, NFYA, MYF6, SP1, NFY, SP4, RFX3, RFX4, RFX1, KLF4, ATF1, RFX5, KLF16
	Osteoblast Differentiated	20	SP3, RARG, KLF14, KLF12, KLF7, NFIC, SP8, CREB1, NFYA, MYF6, SP1, NFY, SP4, RFX3, RFX4, RFX1, KLF4, ATF1, RFX5, KLF16
	Skeletal Muscle Cell Differentiated Into Myotube	19	SP3, RARG, KLF14, KLF12, KLF7, SP8, CREB1, NFYA, MYF6, SP1, NFY, SP4, RFX3, RFX4, RFX1, KLF4, ATF1, RFX5, KLF16
	Smooth Muscle	6	CREB1, NFYA, NFY, ATF1, SP1, SP4
Female Reproductive System	Ovary	7	CREB1, NFYA, NFY, ATF1, SP1, SP4, KLF16
	Uterus	20	SP3, RARG, KLF14, KLF12, KLF7, NFIC, SP8, CREB1, NFYA, MYF6, SP1, NFY, SP4, RFX3, RFX4, RFX1, KLF4, ATF1, RFX5, KLF16
	Cervix	7	CREB1, NFYA, NFY, ATF1, SP1, SP4, KLF16
Male Reproductive System	Prostate	6	CREB1, NFYA, NFY, ATF1, SP1, SP4
	Testis	16	SP3, RARG, KLF14, KLF12, KLF7, NFIC, SP8, MYF6, SP1, SP4, RFX3, RFX4, RFX1, KLF4, RFX5, KLF16
	Seminal Vesicle	21	SP3, RARG, KLF14, KLF12, KLF7, NFIC, SP8, CREB1, NFYA, AP1, MYF6, SP1, NFY, SP4, RFX3, RFX4, RFX1, KLF4, ATF1, RFX5, KLF16

	Ductus Deferens	7	CREB1, NFYA, NFY, ATF1, SP1, SP4, KLF16
	Epididymis	2	CREB1, NFYA
	Penis	1	IKZF2
Endocrine System	Salivary Gland	7	CREB1, NFYA, NFY, ATF1, SP1, SP4, KLF16
	Parotid Gland	4	CREB1, NFYA, NFY, ATF1
	Thyroid	16	SP3, RARG, KLF14, KLF12, KLF7, NFIC, SP8, MYF6, SP1, SP4, RFX3, RFX4, RFX1, KLF4, RFX5, KLF16
Immune System	Tonsil	2	SP1, SP4
	Thymus	22	SP3, RARG, KLF14, KLF12, KLF7, NFIC, SP8, CREB1, NFYA, IKZF2, MYF6, SP1, NFY, SP4, RFX3, ZNF219, RFX4, RFX1, KLF4, ATF1, RFX5, KLF16
	Lymph Node	7	CREB1, NFYA, NFY, ATF1, SP1, SP4, KLF16

Supplementary Table S4: Confidence Score of GDF11 and the Interaction Protein in STRING Network

Source	Target	Weight
GDF11	ACVR1B	1
GDF11	ACVR1C	1
GDF11	ACVR2A	1
GDF11	ACVR2B	1
GDF11	TGFBR1	1
GDF11	WFIKKN2	1
GDF11	ATOH7	1
GDF11	WIF1	1
GDF11	PCSK5	1
GDF11	MNX1	1

ACVR1B	SMAD2	1
ACVR1B	INHBE	1
ACVR1B	SMAD4	1
ACVR1B	INHBA	1
ACVR1B	SMAD3	1
ACVR1B	INHBB	1
ACVR1B	ACVR2B	1
ACVR1B	ACVR2A	1
ACVR1B	TDGF1	1
ACVR1B	LEFTY2	1
ACVR1C	SMAD2	1
ACVR1C	INHBB	1
ACVR1C	TDGF1	1
ACVR1C	INHBA	1
ACVR1C	SMAD3	1
ACVR1C	SMAD4	1
ACVR1C	GDF1	1
ACVR1C	ACVR2B	1
ACVR1C	ACVR2A	1
ACVR1C	INHBE	1
ACVR2A	INHBA	1
ACVR2A	INHBB	1
ACVR2A	BMP2	1
ACVR2A	INHBE	1
ACVR2A	BMP7	1
ACVR2A	SMAD2	1
ACVR2A	SMAD4	1
ACVR2A	SMAD5	1
ACVR2A	ACVR1B	1
ACVR2A	ACVR1	1
ACVR2B	SMAD2	1
ACVR2B	INHBA	1
ACVR2B	INHBB	1
ACVR2B	BMP2	1
ACVR2B	INHBE	1
ACVR2B	LEFTY2	1
ACVR2B	LEFTY1	1
ACVR2B	SMAD4	1
ACVR2B	SMAD5	1
ACVR2B	ACVR1B	1
TGFBR1	SMAD2	0.997
TGFBR1	TGFB1	0.996
TGFBR1	SMAD3	0.995
TGFBR1	SMAD7	0.994
TGFBR1	SMAD4	0.99
TGFBR1	SMAD6	0.984
TGFBR1	TGFB3	0.982
TGFBR1	TGFBR2	0.982
TGFBR1	FKBP1A	0.981
TGFBR1	TGFB2	0.981

WFIKKN2	MSTN	0.823
WFIKKN2	NTSR1	0.758
WFIKKN2	GDF11	0.743
ATOH7	TCF4	0.789
ATOH7	TCF3	0.776
ATOH7	TCF12	0.76
ATOH7	POU4F2	0.76
ATOH7	GDF11	0.72
ATOH7	PIWIL4	0.7
WIF1	WNT3A	0.97
WIF1	WNT5A	0.969
WIF1	WNT1	0.965
WIF1	WNT4	0.964
WIF1	FZD1	0.948
WIF1	LRP6	0.946
WIF1	WNT9A	0.933
WIF1	WNT7A	0.917
WIF1	WNT11	0.915
WIF1	WNT7B	0.877
PCSK5	NGF	0.930
PCSK5	LPL	0.906
PCSK5	GPIHBP1	0.900
PCSK5	REN	0.852
PCSK5	KRT6B	0.839
PCSK5	LIPN	0.791
PCSK5	PGA5	0.749
PCSK5	BACE1	0.746
PCSK5	CTSD	0.744
MNX1	GDPD5	0.726
MNX1	GDF11	0.719
ISL2	GDPD5	1
ISL1	GDPD5	1
ISL1	NEUROG3	1
PBX1	NEUROG3	1
PBX1	ISL1	1
LPL	GPIHBP1	1
LPL	REN	1
WNT3A	WNT5A	1
WNT3A	WNT1	1
WNT3A	WNT4	1
WNT3A	FZD1	1
WNT3A	LRP6	1
WNT3A	WNT9A	1
WNT3A	WNT7A	1
WNT3A	WNT11	1
WNT3A	WNT7B	1
WNT5A	WNT3A	1
WNT5A	WNT1	1
WNT5A	WNT4	1
WNT5A	FZD1	1

WNT5A	LRP6	1
WNT5A	WNT9A	1
WNT5A	WNT7A	1
WNT5A	WNT11	1
WNT5A	WNT7B	1
WNT1	WNT3A	1
WNT1	WNT5A	1
WNT1	WNT4	1
WNT1	FZD1	1
WNT1	LRP6	1
WNT1	WNT9A	1
WNT1	WNT7A	1
WNT1	WNT11	1
WNT1	WNT7B	1
WNT4	WNT3A	1
WNT4	WNT5A	1
WNT4	WNT1	1
WNT4	FZD1	1
WNT4	LRP6	1
WNT4	WNT9A	1
WNT4	WNT7A	1
WNT4	WNT11	1
WNT4	WNT7B	1
FZD1	WNT3A	1
FZD1	WNT5A	1
FZD1	WNT1	1
FZD1	WNT4	1
FZD1	LRP6	1
FZD1	WNT9A	1
FZD1	WNT7A	1
FZD1	WNT11	1
FZD1	WNT7B	1
LRP6	WNT3A	1
LRP6	WNT5A	1
LRP6	WNT1	1
LRP6	WNT4	1
LRP6	FZD1	1
LRP6	WNT9A	1
LRP6	WNT7A	1
LRP6	WNT11	1
LRP6	WNT7B	1
WNT9A	WNT3A	1
WNT9A	WNT5A	1
WNT9A	WNT1	1
WNT9A	WNT4	1
WNT9A	FZD1	1
WNT9A	LRP6	1
WNT9A	WNT7A	1
WNT9A	WNT11	1
WNT9A	WNT7B	1

WNT7A	WNT3A	1
WNT7A	WNT5A	1
WNT7A	WNT1	1
WNT7A	WNT4	1
WNT7A	FZD1	1
WNT7A	LRP6	1
WNT7A	WNT9A	1
WNT7A	WNT11	1
WNT7A	WNT7B	1
WNT11	WNT3A	1
WNT11	WNT5A	1
WNT11	WNT1	1
WNT11	WNT4	1
WNT11	FZD1	1
WNT11	LRP6	1
WNT11	WNT9A	1
WNT11	WNT7A	1
WNT11	WNT7B	1
WNT7B	WNT3A	1
WNT7B	WNT5A	1
WNT7B	WNT1	1
WNT7B	WNT4	1
WNT7B	FZD1	1
WNT7B	LRP6	1
WNT7B	WNT9A	1
WNT7B	WNT7A	1
WNT7B	WNT11	1
DICER1	PIWIL1	1
DICER1	PIWIL4	1
TCF4	TCF3	1
TCF3	TCF12	1
TCF12	TCF4	1
POU4F2	VSX2	1
POU4F2	PAX6	1
VSX2	PAX6	1
SMAD2	TGFB1	1
SMAD2	SMAD3	1
SMAD2	SMAD7	1
SMAD2	SMAD4	1
SMAD2	SMAD6	1
SMAD2	TGFB3	1
SMAD2	TGFBR2	1
SMAD2	FKBP1A	1
TGFB1	SMAD3	1
TGFB1	SMAD7	1
TGFB1	SMAD4	1
TGFB1	SMAD6	1
TGFB1	TGFB3	1
TGFB1	TGFBR2	1
TGFB1	FKBP1A	1

SMAD3	SMAD7	1
SMAD3	SMAD4	1
SMAD3	SMAD6	1
SMAD3	TGFB3	1
SMAD3	TGFBR2	1
SMAD3	FKBP1A	1
SMAD7	SMAD4	1
SMAD7	SMAD6	1
SMAD7	TGFB3	1
SMAD7	TGFBR2	1
SMAD4	SMAD6	1
SMAD4	TGFB3	1
SMAD4	TGFBR2	1
SMAD6	TGFB3	1
SMAD6	TGFBR2	1
SMAD6	FKBP1A	1
TGFB3	TGFBR2	1
TGFB3	FKBP1A	1
TGFBR2	FKBP1A	1
TGFB2	SMAD2	1
TGFB2	TGFB1	1
TGFB2	SMAD3	1
TGFB2	SMAD7	1
TGFB2	SMAD4	1
TGFB2	SMAD6	1
TGFB2	TGFB3	1
TGFB2	TGFBR2	1
BMP2	SMAD4	1
BMP2	SMAD5	1
BMP2	SMAD2	1
SMAD4	SMAD2	1
SMAD4	INHBA	1
SMAD4	INHBB	1
SMAD4	INHBE	1
SMAD4	LEFTY2	1
SMAD4	LEFTY1	1
SMAD4	SMAD5	1
SMAD4	ACVR1B	1
LEFTY1	SMAD2	1
LEFTY1	INHBE	1
LEFTY1	ACVR1B	1
INHBB	INHBA	1
INHBB	ACVR1B	1
INHBB	SMAD2	1
INHBA	ACVR1B	1
INHBA	SMAD2	1
INHBA	SMAD5	1
ACVR1B	BMP2	1
ACVR1B	LEFTY2	1
ACVR1B	INHBE	1

ACVR1B	SMAD5	1
ACVR1B	SMAD2	1
LEFTY2	SMAD5	1
LEFTY2	INHBE	1
LEFTY2	SMAD2	1
INHBE	SMAD5	1
INHBE	SMAD2	1
SMAD5	SMAD2	1
SMAD5	INHBB	1
INHBE	SMAD2	1
INHBE	TDGF1	1
INHBE	SMAD3	1
INHBE	SMAD4	1
INHBE	ACVR2B	1
INHBE	ACVR2A	1
ACVR2A	INHBB	1
ACVR2A	TDGF1	1
ACVR2A	INHBA	1
ACVR2A	SMAD3	1
ACVR2A	SMAD4	1
ACVR2A	GDF1	1
ACVR2A	ACVR2B	1
INHBB	SMAD2	1
INHBB	TDGF1	1
INHBB	INHBA	1
INHBB	SMAD3	1
INHBB	SMAD4	1
INHBB	ACVR2B	1
INHBA	SMAD2	1
INHBA	TDGF1	1
INHBA	SMAD3	1
INHBA	SMAD4	1
INHBA	ACVR2B	1
GDF1	SMAD2	1
GDF1	TDGF1	1
GDF1	SMAD3	1
GDF1	SMAD4	1
GDF1	ACVR2B	1
TDGF1	SMAD2	1
TDGF1	SMAD3	1
TDGF1	SMAD4	1
TDGF1	ACVR2B	1
SMAD4	SMAD2	1
SMAD4	SMAD3	1
SMAD4	ACVR2B	1
SMAD3	ACVR2B	1
TDGF1	SMAD2	1
TDGF1	INHBE	1
TDGF1	SMAD4	1
TDGF1	INHBA	1

TDGF1	SMAD3	1
TDGF1	INHBB	1
TDGF1	ACVR2B	1
TDGF1	ACVR2A	1
TDGF1	LEFTY2	1
LEFTY2	SMAD2	1
LEFTY2	INHBE	1
LEFTY2	SMAD4	1
LEFTY2	SMAD3	1
LEFTY2	INHBB	1
LEFTY2	ACVR2B	1
LEFTY2	ACVR2A	1
SMAD4	SMAD2	1
SMAD4	INHBE	1
SMAD4	INHBA	1
SMAD4	SMAD3	1
SMAD4	INHBB	1
SMAD4	ACVR2B	1
SMAD4	ACVR2A	1
INHBA	SMAD2	1
INHBA	SMAD3	1
INHBA	INHBB	1
INHBA	ACVR2B	1
INHBA	ACVR2A	1
INHBB	SMAD2	1
INHBB	INHBE	1
INHBB	SMAD3	1
INHBB	ACVR2B	1
INHBB	ACVR2A	1
ACVR2A	SMAD2	1
ACVR2A	INHBE	1
ACVR2A	SMAD3	1
ACVR2A	ACVR2B	1
ACVR2B	SMAD2	1
ACVR2B	INHBE	1
ACVR2B	SMAD3	1
SMAD2	INHBE	1
SMAD2	SMAD3	1
SMAD3	INHBE	1
